# Risk and protective factors associated with depressive symptoms in older adults with visual impairment: a systematic review

**DOI:** 10.1093/geront/gnag133

**Published:** 2026-06-13

**Authors:** Haruno Suzuki, Heather Leutwyler, Jacqueline M Torres, Kenneth Covinsky, Margaret Wallhagen

**Affiliations:** Department of Physiological Nursing, University of California, San Francisco, San Francisco, California, United States; Department of Physiological Nursing, University of California, San Francisco, San Francisco, California, United States; Department of Epidemiology and Biostatistics, University of California, San Francisco, San Francisco, California, United States; Division of Geriatrics, University of California, San Francisco, San Francisco, California, United States; Department of Physiological Nursing, University of California, San Francisco, San Francisco, California, United States

**Keywords:** Depressive symptoms, Older adults, Protective factor, Risk factor, Systematic review, Visual impairment

## Abstract

**Background and Objectives:**

This systematic review aimed to synthesize risk and protective factors associated with depressive symptoms in older adults with visual impairment.

**Research Design and Methods:**

We included quantitative studies that examined factors associated with depressive symptoms in adults aged 60 or older with visual impairment. Five electronic bibliographic databases (PubMed, Web of Science, Embase, PsycINFO, and CINAHL Complete) were searched in November 2025 in collaboration with medical librarians. Two reviewers independently screened titles, abstracts, and full texts, and any discrepancy was resolved by a third reviewer. The National Heart, Lung, and Blood Institute’s Quality Assessment Tool was used to assess risk of bias in individual studies. The included studies were qualitatively synthesized to summarize the evidence.

**Results:**

Out of 14,963 records identified, 13 studies met the eligibility criteria. These studies comprised 15 reports, including 11 cross-sectional and 4 longitudinal designs. Most reports were assessed as “fair” quality due to a lack of sample size justification and the inability to establish temporality. Risk factors included older age, being widowed, severe visual impairment, severe self-reported visual difficulty, poor self-rated health, and greater ADL/IADL limitations. Protective factors included better adaptive coping strategies, a greater level of social support, and the use of rehabilitation services and optical aids.

**Discussion and Implications:**

Findings suggest adaptive coping strategies and social support may be key modifiable protective factors for depressive symptoms among older adults with visual impairment. Findings may guide novel interventions enhancing psychological adaptation to visual impairment and maintaining functional independence.

Visual impairment is one of the most common functional impairments among older adults (GBD 2019 Ageing Collaborators, 2022). Visual impairment disproportionately affects older adults; most cases of blindness or visual impairment are in adults aged 50 and older (World Health Organization [WHO], 2026). Worldwide, 600 million people had distance visual impairment or blindness in 2020, and this number is projected to reach 895 million by 2050, as the global population ages (GBD 2019 Blindness and Vision Impairment Collaborators & Vision Loss Expert Group of the Global Burden of Disease Study, 2021). Prior meta-analyses identified strong evidence that visual impairment is associated with poorer quality of life (QOL) (Assi et al., 2021), functional limitations (Rahmati et al., 2025), cognitive decline (Shang et al., 2021), and increased mortality (Ehrlich et al., 2021). Given the growing number of older adults with visual impairment globally, promoting optimal health becomes increasingly important in this population.

Depressive symptoms are a prevalent comorbidity in older adults with visual impairment, with several meta-analyses reporting prevalences of depressive symptoms ranging from 25.0% to 27.0% (Parravano et al., 2021; Virgili et al., 2022). Although these prevalences are comparable to those observed in the general older population (28.4%–31.7%; Hu et al., 2022; Zenebe et al., 2021), the pathways leading to depressive symptoms in older adults with visual impairment may involve unique, vision-related stressors. These vision-specific stressors include difficulties in psychological adjustment to irreversible visual impairment, lifestyle changes due to reduced vision functioning, and a loss of independence, as well as multimorbidity, reduced social interactions, stigma, and limited access to mental health services (Bennion et al., 2012; Donato et al., 2024; Heesterbeek et al., 2017; Kumar et al., 2022; Nyman et al., 2012; Xiong et al., 2023). Addressing depressive symptoms among older population with visual impairment is critical because late-life depression contributes to accelerated cognitive decline (John et al., 2019), increased suicidal behaviors (Yang et al., 2025), and elevated mortality risk (Fang et al., 2025; Wei et al., 2019). Depressive symptoms involve persistent depressed mood, loss of interest or pleasure, and accompanying changes in sleep, appetite, or energy (American Psychiatric Association [APA], 2022). Late-life depression has specific characteristics, with older adults more likely to endorse somatic symptoms (e.g., decreased appetite, insomnia, fatigue), cognitive changes similar to dementia (e.g., memory impairment, poor concentration), and loss of interest rather than depressed mood compared to younger adults (Kok & Reynolds, 2017). Although diagnostic criteria for major depressive disorder are outlined in the Diagnostic and Statistical Manual of Mental Disorders, Fifth Edition, Text Revision (DSM-5-TR) (APA, 2022), many older adults experience depressive symptoms that do not meet full diagnostic thresholds yet limit their daily functioning and QOL. Moreover, because comorbid somatic or cognitive symptoms overlap with depressive symptoms due to common late-life comorbidities (e.g., cancer, dementia), the assessment of late-life depression poses methodological challenges for precise estimation (Haigh et al., 2018). These challenges may lead to underdiagnosis of depressive symptoms and delayed treatment among older adults. Since early detection and treatment are essential to address the prognosis of depressive symptoms, knowledge of risk and protective factors may help identify high-risk groups and offer personalized preventative approaches.

Given the multifactorial nature of depressive symptoms in late life, this systematic review applies the Disablement Process Model (DPM) (Verbrugge & Jette, 1994) as a theoretical framework. The DPM conceptualizes the disablement process as follows: (1) a sequential process starting when older adults experience visual impairment, followed by functional limitations (e.g., restrictions to see standard prints or people’s face) and then disabilities (e.g., difficulties in doing activities of daily living or social participation); (2) both social and environmental facilitators and barriers affect health outcomes; and (3) predisposing risk factors include sociodemographic, biological, physical, psychological, behavioral, social, and environmental domains. This model provides a holistic framework to map the relevant factors that may accelerate or buffer depressive symptoms among older adults with visual impairment.

Prior systematic reviews and meta-analyses in the general older population identified a wide range of risk factors associated with depressive symptoms, that include older age, female sex/gender, lower educational attainment, bereavement, poor self-rated health, functional disability, chronic diseases, sleep disturbances, cognitive impairment, loneliness, and stressful life events (Maier et al., 2021; Silva et al., 2023; Wu et al., 2022; Zenebe et al., 2021). Protective factors include physical activity, greater levels of social support, and psychological resilience (Maier et al., 2021; Silva et al., 2023; Wermelinger Ávila et al., 2017; Zenebe et al., 2021).

Focusing on older adults with visual impairment, only one systematic review (Ribeiro et al., 2015) examined physical health factors, such as functional disability and chronic diseases, as risk factors for depressive symptoms. However, this review had several limitations: (a) it focused on only physical health factors and did not comprehensively examine sociodemographic or psychosocial factors; (b) it presented mixed results from populations with and without visual impairment; and (c) it did not clearly specify whether confounding factors were adjusted for in multivariable analyses in included studies. Because this systematic review was published in 2015, several additional reports have been published (Gouliopoulos et al., 2026; Palagyi et al., 2016; Schilling et al., 2016; Yimer et al., 2021; Zhou, 2025), including those conducted in under-represented regions, such as sub-Saharan Africa (Yimer et al., 2021). However, to the best of our knowledge, there is no systematic review to comprehensively synthesize risk and protective factors associated with depressive symptoms among older adults with visual impairment.

To fill this knowledge gap, this systematic review aims to summarize risk and protective factors associated with depressive symptoms among older adults with visual impairment. The findings may contribute to the latest science in gerontology in three significant ways: (a) informing clinical guidelines for early identification and tailored interventions for depressive symptoms; (b) guiding public health policies to improve mental health care access in ophthalmologic and low vision rehabilitation settings; and (c) providing theoretical implications to advance conceptual frameworks on late-life mental health.

## Method

This systematic review was conducted in accordance with the Preferred Reporting Items for Systematic Reviews and Meta-Analyses (PRISMA) 2020 Statement (Page et al., 2021; Supplementary Material Table 1). The protocol of this systematic review was registered with the International Prospective Register of Systematic Reviews (PROSPERO: CRD42024602520).

### Eligibility criteria

Table 1 summarizes the inclusion and exclusion criteria of this review based on the PECOS framework (i.e., populations, exposures, comparators, outcome(s) of interest, and study designs/type) (Schardt et al., 2007). We included peer-reviewed journal articles which were available in full-text written in English. We excluded review papers, protocols, theoretical papers, case reports, editorials, letters, expert forum papers, gray literature, conference papers, and dissertations.

**Table 1:** Eligibility criteria based on PECOS framework.

		Inclusion criteria	Exclusion criteria
*P*	Populations	Older adults (aged 60 years or older)^a^ with visual impairment^b^^,^^c^	Adults with visual impairment who are younger than 60 years of age Older adults with congenital visual impairment Older adults with dual sensory loss Older adults with self-reported visual difficulty or functional vision rather than visual impairment using objective measures of visual acuity^b^^,^^c^
*E*	Exposures	Studies that examined risk and/or protective factors associated with depressive symptoms (e.g., sociodemographic, physical, and psychosocial factors) using multivariable statistical analyses to control for confounding factors^d^	Studies that used the presence of visual impairment as the primary exposure (i.e., comparing older adults with and without visual impairment) Studies that conducted only univariate (i.e., unadjusted) regression analyses
*C*	Comparators	Not applicable	Not applicable
*O*	Outcome(s)	Depressive symptoms^e^^,^^f^	None
*S*	Study designs/types	Cohort studies Cross-sectional studies Case-control studies Randomized controlled trials^g^ Quasi-experimental studies^g^ Mixed methods studies^g^	Qualitative studies

a Older adults were defined as people aged 60 or older (World Health Organization, 2025a).

b Visual impairment was defined as a significant limitation of visual capability that cannot be corrected by refractive correction, medication, or surgery and in which visual acuity is worse than 20/40 in the better-seeing eye (National Library of Medicine, 2024; World Health Organization, 2024).

c Visual impairment was necessary to be documented objective measures of visual acuity (e.g., Snellen chart, the Logarithm of the Minimum Angle of Resolution (logMAR) chart).

d Multivariable regression analyses or equivalent statistical methods (e.g., generalized linear mixed models) within older adults with visual impairment was necessary to control for confounding factors.

e The definition of depressive symptoms follows the criteria of major depressive disorders and subthreshold depression in the Diagnostic and Statistical Manual for Mental Disorders, Fifth Edition, Text Revision (DSM-5-TR; American Psychiatric Association, 2022).

f Depressive symptoms were necessary to be documented using diagnostic criteria, clinical evaluation, or valid and reliable self-report measures. There were no restrictions by types, duration, or severity of depressive symptoms.

g Randomized controlled trials, quasi-experimental studies, and mixed method studies were included if they assessed visual impairment, depression, and risk/protective factors associated with depressive symptoms and used baseline data before interventions.

Older adults were defined as people aged 60 or older (WHO, 2025a). Visual impairment was defined as a significant limitation of visual capability that cannot be corrected by refractive correction, medication, or surgery and in which visual acuity is worse than 20/40 in the better-seeing eye (National Library of Medicine, 2024; WHO, 2024). We included studies that documented visual impairment using objective measures of visual acuity (e.g., Snellen chart, the Logarithm of the Minimum Angle of Resolution (logMAR) chart). Studies that measured only self-reported visual difficulty or functional vision without objective measures of visual acuity were excluded. The definition of depressive symptoms follows the criteria of major depressive disorders and subthreshold depression in the DSM-5-TR (APA, 2022). Multivariable regression analyses within older adults with visual impairment was necessary to control for confounding factors.

### Information sources and search strategy

In collaboration with two medical librarians, literature search strategies were developed using medical subject headings (MeSH) terms and various text words related to risk and protective factors associated with depressive symptoms in older adults with visual impairment. The following five bibliographic databases were searched: PubMed, Embase, Web of Science, PsycINFO, and CINAHL Complete. Supplementary Material Table 2 shows examples of comprehensive search strategies for each database. The original search was conducted on November 17, 2024. The updated search using the same search strategies was re-run on November 20, 2025, before completing the data extraction and final analyses. Studies available online by November 20, 2025, were eligible for inclusion, including those subsequently assigned to formal journal issues in 2026. No date limits were applied to the searches to increase the sensitivity of this review.

### Selection process

**Figure 1:**
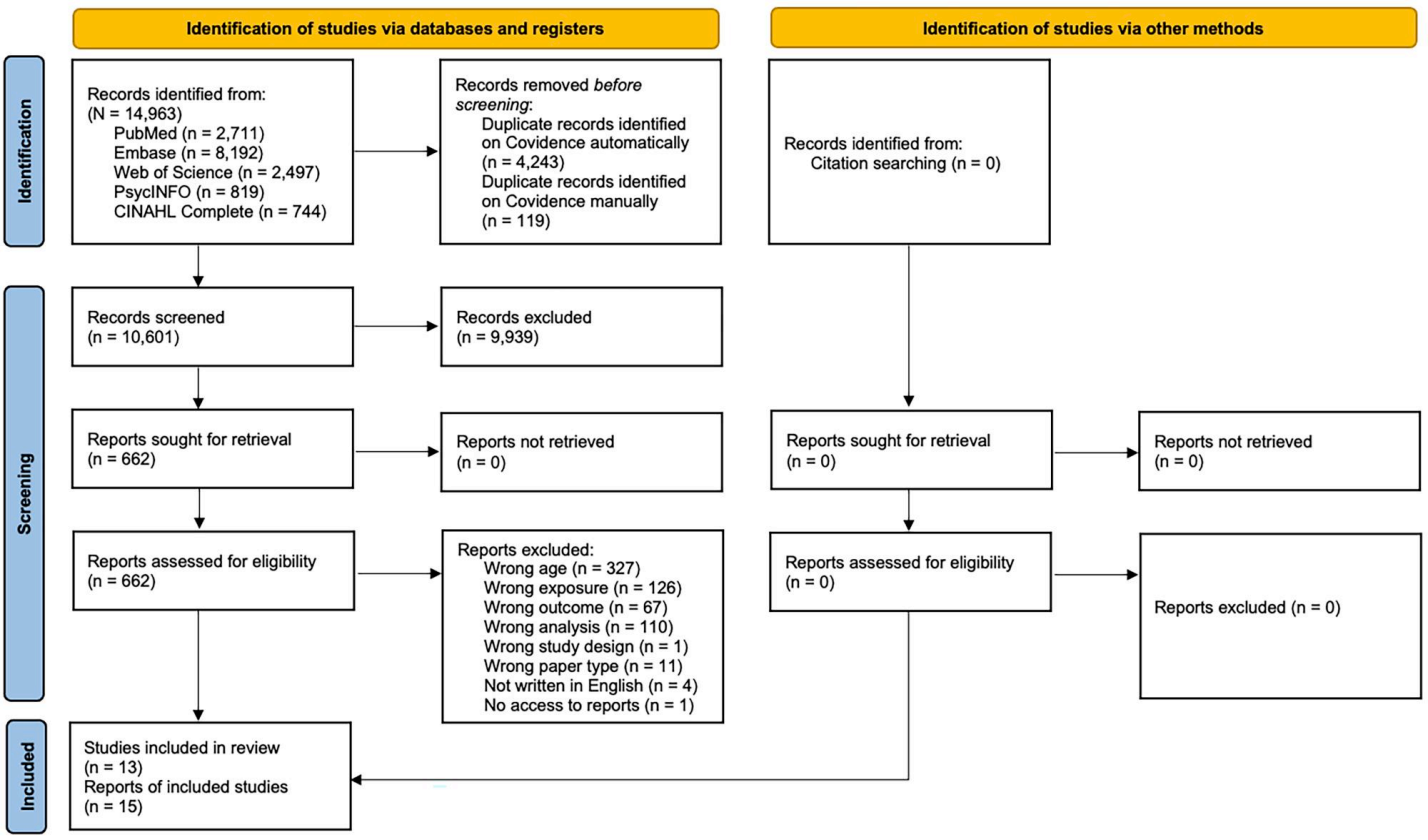
Preferred Reporting Items for Systematic Reviews and Meta-Analyses (PRISMA) 2020 flow diagram to screen studies.

All searched literature data were uploaded to Zotero reference management software (Corporation for Digital Scholarship, Vienna, VA). All retrieved records were then imported into Covidence systematic review software (Veritas Health Innovation Ltd, Melbourne, Victoria, Australia), which supports collaboration with reviewers during the selection process. Duplicates of retrieved records were removed on Covidence. Using Covidence, two reviewers independently screened the titles and abstracts based on the pre-specified eligibility criteria (Table 1). To ensure consistency in the review process, before starting the full text screening, the reviewers conducted a pilot study that randomly selected 30 reports and discussed the procedure for the full text screening. Then, two independent reviewers reviewed the full texts of the selected reports to finalize the number of studies to be reviewed. Full-text reports were collected to ascertain the inclusion criteria and assess any uncertainty in the reports. Full text reports that meet the pre-specified inclusion criteria were retrieved. The two reviewers resolved discrepancies through discussion with a third reviewer. The percentage of agreement was 91.9% (Cohen’s Kappa coefficient = 0.38) for the title and abstract screening and 83.9% (Cohen’s Kappa coefficient = 0.45) for the full text screening. Following the PRISMA 2020 statement (Page et al., 2021), the PRISMA 2020 flow diagram (Figure 1) shows the selection process and the reasons for excluding studies. If multiple reports were derived from the same study cohort, they were counted as a single study to avoid double-counting. A first reviewer contacted study authors if needed to clarify the unclear points for eligibility criteria.

### Data collection process and data items

The data from each study were extracted based on pre-specified review criteria. Our pre-specified review criteria include: author(s); year of publication; study aims; study design; sample size; study region/country; participants’ characteristics (e.g., age, sex/gender, race/ethnicity, education, income level, living arrangement, marital status, study settings, physical function, cognitive function, comorbidities); type, severity, and duration of visual impairment; causes of visual impairment; occurrence, type, severity, and duration of depressive symptoms; study methods (e.g., nomenclature to describe visual impairment; definition(s) of visual impairment and depressive symptoms; instrument(s) of visual impairment and depressive symptoms; theoretical framework(s); covariates that were adjusted for in multivariable models); prevalence or incidence of depressive symptoms; risk and protective factors associated with depressive symptoms; and study’s strengths and limitations.

Data were organized using two tables (i.e., one for cross-sectional reports and one for longitudinal reports). Two reviewers conducted a pilot test to refine data extraction using the data extraction tables and the randomly selected six reports obtained through the selection process. These tables were used to present the synthesized information from retrieved reports. A first reviewer independently extracted the data items, and a second reviewer cross-checked the accuracy of the reviewed reports. Reviewers resolved any conflict by discussion, and a third reviewer arbitrated the unresolved conflict.

### Risk of bias in individual studies

The National Heart, Lung, and Blood Institute’s (NHLBI) Quality Assessment Tool for Observational Cohort and Cross-Sectional Studies (NHLBI, 2021) was used to assess the methodological quality of studies and the risk of bias in individual studies. This quality assessment tool was designed to critically evaluate the possibility of bias in study design, sampling, measurements, and analysis. The rationale for using the NHLBI Quality Assessment Tool for this review is that it applies a single, consistent set of assessment criteria across both cross-sectional and longitudinal studies, unlike other tools (e.g., the JBI Critical Appraisal Tools), enabling a more comparable appraisal of methodological quality across study designs. Additionally, each assessment item is specifically tailored to epidemiological methodology, with explicit attention to selection bias, information bias, measurement bias, and confounding. The tool has 14 items. Following the NHLBI quality assessment guidelines, reviewers selected yes, no, cannot determine, not reported, or not applicable for each item in the tool. If items are selected “no” or indeterminable responses, reviewers considered the potential risk of bias introduced in studies. After completion of assessment, a judgment of “good,” “fair,” or “poor” quality was determined for each study. Two reviewers independently assessed the methodological quality of each study and recorded their assessments in a Word file. Two reviewers resolved any disagreements in rating through discussion with a third reviewer. Before starting the formal critical appraisal, two reviewers conducted a pilot study of 5%–10% of the extracted studies using the tool. All studies that met the inclusion criteria were included in the final review regardless of the quality rating.

A first reviewer created a traffic-light plot to visualize the risk of bias across studies using the robvis software (McGuinness & Higgins, 2021). The level of the risk of bias in individual studies was labeled using a colorblind-friendly palette: “good” (*yellow*), “fair” (*light orange*), and “poor” (*deep orange*). The assessment of the risk of bias in individual studies using the NHLBI Quality Assessment Tool was documented in an Excel file and imported into the robvis online template, and the row for the risk of bias domains was made in accordance with the NHLBI Quality Assessment Tool’s 14 items. Each study’s quality assessment results were chronologically displayed in three types of colors marking as explained above.

### Data synthesis

Because of the heterogeneity in sample characteristics, exposure measures, and outcome measures, we performed a qualitative synthesis of the quantitative studies. We did not conduct a meta-analysis or quantify publication bias due to the heterogeneity across the included studies.

We created a heat map to visualize the distribution of risk and protective factors for depressive symptoms. The purpose of this approach was to identify patterns of evidence across different study designs. The heat map was created based on the direction and statistical significance (defined as two-sided, *p*-value < .05) of the associations reported in the multivariable models. The heat map illustrates the factors categorized as risk factors (pink), protective factors (blue), and factors that did not reach statistical significance (gray). To ensure no data were excluded from our synthesis based on statistical significance, all reported associations were incorporated into the heat map regardless of their *p*-values. Risk factors were defined as factors associated with higher depression scores or odds of being in categorized as depressed or not depressed in the multivariable model. Protective factors were defined as factors associated with lower depression scores or odds of being categorized as depressed or not depressed in the multivariable model. We noted factors of that there was no statistically significant association with depression scores or odds of being categorized as depressed or not depressed in the multivariable model. For each variable (e.g., age, severity of visual impairment), we calculated the frequency of risk factors, protective factors, and factors that did not reach statistical significance. The density of color for each cell reflects the frequency of risk factors, protective factors, and factors that did not reach statistical significance, with a deeper color indicating a higher frequency. We presented the point estimates and 95% confidence intervals or standard errors for all studies in tables to provide context regarding the effect size and precision of each association. The identified factors were classified into five domains: sociodemographic, vision-related, physical health, psychological, and social. The heat map presented the identified factors for all studies combined, as well as for cross-sectional and longitudinal reports separately.

## Results

### Study selection

In this review, “records” refers to citation search results retrieved from five bibliographic databases, “reports” refers to individual published papers, and “studies” refers to unique data sources (e.g., cohorts) which may have yielded more than one report. The initial search retrieved 14,963 records, from which 4,362 duplicates were removed (Figure 1). 10,601 records were screened for title and abstract, and 662 reports were screened for the full texts. After the full-text screening, a total of 13 unique studies (published as 15 reports) met the eligibility criteria and were included for qualitative synthesis. Because Horowitz et al. (2003) and Horowitz et al. (2005a), as well as Horowitz et al. (2005b) and Horowitz et al. (2006), were derived from the same cohorts but examined different exposures, each pair was counted as one study with two reports.

### Risk of bias in individual studies

The results of risk of bias assessments of the 15 reports are reported (Supplementary Material Figure 1 [cross-sectional studies]; Supplementary Material Figure 2 [longitudinal studies]). Only one of the 11 cross-sectional reports received a “good” quality rating and eight received a “fair” rating. Across the 10 reports that received a “fair” rating, the major sources of bias were: (a) lack of sample size justification; (b) exposures were not assessed prior to outcome measurement; and (c) insufficient timeframe to see an effect of exposure. A total of two of the four longitudinal reports received a “good” quality rating, two received a “fair” rating. Across the two reports that received a “fair” rating, the major sources of bias were: (a) lack of sample size justification; (b) exposures not assessed prior to outcome measurement; (c) exposures were not assessed by valid and reliable measures; and (d) exposures were not assessed more than once over time.

### Study characteristics

#### Sociodemographics

The characteristics of the 13 unique studies (15 reports) are shown in Table 2 (cross-sectional) and Table 3 (longitudinal). One study was published in the1990s, five studies in the 2000s, four in the 2010s, and three in the 2020s. Five studies were conducted in the United States, and two in Australia. The six remaining were conducted in Canada, China, Ethiopia, Greece, Hong Kong, and New Zealand. In total, 11 (73.3%) cross-sectional and four (26.7%) longitudinal reports were included. None of these studies evaluated interventions or used mixed methods report. Among four longitudinal reports, all employed a prospective cohort design with follow-up periods ranging from 6 months to 2 years (median = 2 years).

**Table 2:** Study characteristics and identified risk and protective factors associated with depressive symptoms in the included cross-sectional reports (*n *= 11).

**Author(s) (publication year), country** ^a^	Sample characteristics	VI measures (VI definitions)	Depressive symptom measures and definitions (prevalence of depressive symptoms)	Identified risk factors, protective factors, and factors that did not reach statistical significance for depressive symptoms (OR with 95% CI, RR with 95% CI, beta with 95% CI, or *B* with *SE*)^b^	Type of multivariate analyses; covariates that were adjusted for in multivariable models
Kleinschmidt et al. (1995), United States	*N *= 80, ophthalmologic clinic-based convenience sample; 65–91 years old, mean age 76.9 years, 72.5% female, causes of VI: AMD 100.0%	Snellen chart (NR)	30-item GDS ≥11 (26.0%)	*Risk factors:* Greater chance health LOC (beta 0.24, 95% CI NR) *Protective factors:* Greater perceived control (beta −0.65, 95% CI NR) *Factors that did not reach statistical significance:* Powerful others LOC (beta −9.30), chance LOC (beta 0.02), internal LOC (beta −0.04), powerful others Health LOC (beta 0.06), internal Health LOC (beta −0.07) (All 95% CI: NR)	*Type of analyses:* linear regression analysis *Covariates:* powerful others LOC, chance LOC, internal LOC, powerful others Health LOC, chance Health LOC, internal Health LOC, perceived control
Ip et al. (2000), Hong Kong	*N *= 53, long-term care facility-based convenience sample; 60–NR years old, 100.0% female, causes of VI: NR	Snellen chart (BCVA worse than 20/400 in the better eye)	15-item GDS ≥6 (45.2%)	*Risk factors:* Greater ADL limitations^c^ (OR 0.96, 95% CI 0.92–0.99) *Protective factors:* Longer duration of institutionalization at a nursing home (years; OR 0.75, 95% CI 0.59–0.94) *Factors that did not reach statistical significance:* Age (years; OR 1.05, 95% CI 0.92–1.21), duration of VI (years; OR 1.02, 95% CI 0.97–1.08)	*Type of analyses:* logistic regression analysis *Covariates:* age, duration of institutionalization at a nursing home, duration of VI, ADL limitations
Horowitz et al. (2005b), United States	*N *= 584, community-based vision rehabilitation service convenience sample; 65–100 years old, mean age 80.4 years (*SD *= 7.6), 52.7% female, causes of VI: AMD 65.8%, cataract 35.3%, glaucoma 27.1%, DR 14.7%	Snellen chart (NR)	20-item CES-D ≥ 16 (21.2%)	*Risk factors:* Poor self-rated health^d^ (OR 0.76, 95% CI 0.58–1.00), greater ADL/IADL limitations (OR 1.05, 95% CI 1.01–1.08), presence of past negative life events (OR 1.81, 95% CI 1.16–2.83) *Protective factors:* Greater perceived adequacy of social support (OR 0.77, 95% CI 0.66–0.89), higher self-efficacy (OR 0.92, 95% CI 0.86–0.97) *Factors that did not reach statistical significance:* Age (years; OR 1.02, 95% CI 0.99–1.05), gender (female vs male; OR 1.14, 95% CI 0.69–1.87), race (White vs other races; OR 1.39, 95% CI 0.73–2.68), education (years; OR 0.96, 95% CI 0.83–1.10), marital status (currently married vs not; OR 0.88, 95% CI 0.52–1.48), self-reported visual difficulty (OR 0.94, 95% CI 0.86–1.03), number of chronic health conditions (OR 1.06, 95% CI 0.93–1.20), perceived availability of social support (OR 0.92, 95% CI 0.90–1.05)	*Type of analyses:* logistic regression analysis *Covariates:* age, gender, race, education, marital status, self-reported visual difficulty, history of depression, self-rated health, ADL/IADL limitations, number of chronic health conditions, perceived availability of social support, perceived adequacy of social support, self-efficacy, past negative life events
Tolman et al. (2005), United States	*N *= 144, ophthalmologic clinic-based convenience sample; 65–95 years old, mean age 81.6 years (*SD *= 6.2), 72.9% female, causes of VI: AMD 100.0%	Snellen chart (BCVA 20/200 or worse in both eyes)	Short form GDS, definition was NR (NR)	*Risk factors:* None *Protective factors:* Better acceptance of VI^e^ (beta 0.46), positive attitude toward compensation^e^ (beta 0.16), a greater number of using outpatient vision rehabilitation services (beta −0.18) (All 95% CI: NR) *Factors that did not reach statistical significance:* Age (years; beta 0.03), gender (male vs female; beta 0.06), education (years; beta −0.10), negative impact on relationships (beta 0.07) (All 95% CI: NR)	*Type of analyses:* linear regression analysis *Covariates:* age, gender, education, better acceptance of VI, negative impact on relationships, better attitude toward compensation, number of using outpatient vision rehabilitation services
Hayman et al. (2007), New Zealand	*N *= 391, low vision clinic-based convenience sample; 75–NR years old, mean age 83.7 years (*SD *= 4.8), 68.3% female, causes of VI: AMD 84.0%	logMAR (BCVA 20/80 or worse in the better eye)	15-item GDS ≥5 (29.4%)	*Risk factors:* Living at a nursing home (vs living at home; *B* 0.42, *SE* 0.19), greater self-reported visual difficulty^f^ (*B* −0.22, *SE* 0.09), severe state anxiety (*B* 6.06, *SE* 1.31) *Protective factors:* A greater level of physical activity (*B* −0.02, *SE* 0.01), better physical health-related QOL (*B* −0.06, *SE* 0.01), better mental health-related QOL (*B* −0.14, *SE* 0.01) *Factors that did not reach statistical significance:* Age (years; *B* 0.01, *SE* 0.02), gender (male vs female; *B* −0.40, *SE* 0.24), ethnicity (New Zealand European vs other; *B* 0.05, *SE* 0.25), research site (Dunedin vs Auckland; *B* 0.16, *SE* 0.27), severity of VI (*B* 0.12, *SE* 0.11), ADL/IADL limitations (*B* −0.08, *SE* 0.04), fall efficacy (*B* 0.03, *SE* 0.09), ability to stand without support (yes vs no; *B* 0.52, *SE* 0.37), balance level (*B* 0.26, *SE* 0.14), total number of medications (*B* −0.21, *SE* 0.17), use of antidepressants (yes vs no; *B* 0.14, *SE* 0.36)	*Type of analyses:* linear regression analysis *Covariates:* age, gender, ethnicity, living arrangements, research site, severity of VI, self-reported visual difficulty, use of antidepressants, state anxiety, ADL/IADL limitations, fall efficacy, ability to stand without support, balance level, physical activity level, total number of medications
Jivraj et al. (2013), Canada	*N *= 94, ophthalmologic clinic-based convenience sample; 65–NR years old, mean age 80.4 years (*SD *= 6.8), 69.2% female, causes of VI: AMD 100.0%	logMAR, Snellen chart (NR)	20-item CES-D ≥ 16 (21.3%)	*Risk factors:* Living at home alone or living at a nursing home^g^ (vs living at home with others; OR 0.17, 95% CI 0.04–0.76) *Protective factors:* Better vision-related QOL (OR 0.93, 95% CI 0.89–0.97) *Factors that did not reach statistical significance:* Age (decades; OR 0.66, 95% CI 0.24–1.84), sex (male vs female; OR 0.89, 95% CI 0.20–3.97), severity of VI (OR 0.55, 95% CI 0.10–3.02), duration of AMD (years; OR 1.04, 95% CI 0.84–1.29)	*Type of analyses:* logistic regression analysis *Covariates:* age, sex, living arrangements, severity of VI, duration of AMD, vision-related QOL
Holloway et al. (2015), Australia	*N *= 124, community-based low vision rehabilitation and eye-care service convenience sample; 60–94 years old, mean age 77.0 years (*SD *= 9.1), 71.0% female, causes of VI: AMD 51.2%, glaucoma 17.9%, DR 8.9%, cataract 2.4%, other 19.5%	logMAR, Snellen chart (BCVA worse than 20/40 in the better eye)	2-item PHQ ≥3 (37.1%)	*Risk factors:* Severe VI (vs mild VI; OR 7.41, 95% CI 1.59–34.62) *Protective factors:* None *Factors that did not reach statistical significance:* Age (years; OR 0.95, 95% CI 0.89–1.00), sex (female vs male; OR 0.49, 95% CI 0.12–1.92), depression treatment status (current vs never; OR 16.50, 95% CI 0.09–8.28), perceived need for psychological help (OR 1.37, 95% CI 0.81–2.32)	*Type of analyses:* logistic regression analysis *Covariates:* age, sex, severity of VI, history of depression, depression treatment status, perceived need for psychological help
Palagyi et al. (2016), Australia	*N *= 329, ophthalmologic clinic-based convenience sample awaiting cataract surgery; 65–NR years old, mean age 75.7 years (*SD *= 5.3), 55.3% female, causes of VI: cataract 100.0%	ETDRS (NR)	5-item GDS ≥2 (9.7%)	*Risk factors:* Greater self-reported visual difficulty (RR 1.08, 95% CI 1.01–1.14), a greater number of comorbidities (RR 1.10, 95% CI 1.02–1.19) *Protective factors:* Better health-related QOL (RR 0.94, 95% CI 0.90–0.98) *Factors that did not reach statistical significance:* None	*Type of analyses:* Poisson regression analysis *Covariates:* age, sex, self-reported visual difficulty, number of comorbidities, health-related QOL
Yimer et al. (2021), Ethiopia	*N *= 412, ophthalmologic clinic-based convenience sample; 60–NR years old, mean age 72.6 years (*SD *= 6.5), 36.4% female, causes of VI: cataract 33.2%, glaucoma 31.3%, DR 7.8%, other 27.7%	Snellen chart (visual acuity 20/30 or worse)	15-item GDS ≥6 (26.7%)	*Risk factors:* Widowed (vs married; OR 3.17, 95% CI 1.71–5.91), single/divorced (vs married; OR 2.70, 95% CI 1.35–5.38), severe VI (vs mild VI; OR 2.63, 95% CI 1.73–6.63), >5 years duration of VI (vs 2≥ years; OR 3.15, 95% CI 1.60–6.19) *Protective factors:* A greater level of social support^h^ (vs lower level; OR 4.34, 95% CI 1.84–10.24) *Factors that did not reach statistical significance:* Age (>80 vs 60–65 years; OR 1.67, 95% CI 0.63–4.48), sex (female vs male; OR 1.48, 95% CI 0.87–2.49), education (no formal education vs college and above; OR 1.36, 95% CI 0.58–3.18), residence area (rural vs urban; OR 1.57, 95% CI 0.83–2.65), history of mental illness (yes vs no; OR 1.76, 95% CI 0.93–3.35), current substance use (yes vs no; OR 1.36, 95% CI 0.74–2.59)	*Type of analyses:* logistic regression analysis *Covariates:* age, sex, education, marital status, residence area, severity of VI, duration of VI, history of mental illness, current substance use, level of social support
Zhou (2025), China	*N *= 197, ophthalmologic clinic-based convenience sample; 60–NR years old, 57.4% female, causes of VI: cataract 100.0%	Decimal visual acuity chart (NR)	20-item SDS ≥50 (44.7%)	*Risk factors:* Older age (years; OR 1.05, 95% CI 1.01–1.10), unhealthy caregiver health status (vs healthy; OR 1.97, 95% CI 1.01–3.83), presence of diabetes (OR 2.40, 95% CI 1.05–5.46) *Protective factors:* None *Factors that did not reach statistical significance:* None	*Type of analyses:* logistic regression analysis *Covariates:* age, severity of VI, caregiver health status, presence of diabetes, history of stroke
Gouliopoulos et al. (2026), Greece	*N *= 146, ophthalmologic clinic-based convenience sample; 65–93 years old, causes of VI: AMD 100.0%	logMAR (NR)	20-item SDS ≥50 (NR)	*Risk factors:* Widowed (vs married; OR 9.73, 95% CI 1.72–55.01), wet-type AMD (vs dry; OR 5.28, 95% CI 1.48–19.23), a history of cataract surgery (vs no history; OR 5.90, 95% CI 2.08–16.17), *Protective factors:* Presence of smoking behaviors (vs absence; OR 0.06, 95% CI 0.01–0.37) *Factors that did not reach statistical significance:* Age (years; OR 0.90, 95% CI 0.80–1.01), sex (female vs male; OR 3.05, 95% CI 0.88–10.58), educational attainment (secondary/diploma vs primary; OR 1.35, 95% CI 0.51–3.59), financial status (good/moderate vs bad; OR 0.74, 95% CI 0.24–2.30), severity of VI (OR 3.02, 95% CI 0.60–15.19), duration of AMD (years; OR 1.20, 95% CI 0.99–1.44), presence of diabetes (yes vs no; OR 1.09, 95% CI 0.27–4.36), presence of hypertension (yes vs no; OR 1.31, 95% CI 0.41–4.19), history of cancer (yes vs no; OR 0.40, 95% CI 0.01–17.60), use of oral antioxidant supplements (yes vs no; OR 0.47, 95% CI 0.14–1.61)	*Type of analyses:* logistic regression analysis *Covariates:* age, sex, marital status, educational attainment, financial status, severity of VI, type of AMD, duration of AMD, presence of diabetes, presence of hypertension, history of cancer, history of cataract surgery, smoking behaviors, use of oral antioxidant supplements

*Note*. ADL = activities of daily living; AMD = age-related macular degeneration; *B* = unstandardized coefficient; BCVA = best-corrected visual acuity; beta–standardized coefficient; CES-D = Center for Epidemiologic Studies Depression Scale; CI = confidence interval; DR = diabetic retinopathy; ETDRS = Early Treatment Diabetic Retinopathy Study; GDS = Geriatric Depression Scale; IADL = instrumental activities of daily living; LOC = locus of control; logMAR = logarithm of the Minimum Angle of Resolution; NR = not reported; OR = odds ratio; PHQ = Patient Health Questionnaire; QOL = quality of life; RR = rate ratio; *SD* = standard deviation; SDS = Zung Self-Rating Depression Scale; *SE* = standard error; VI = visual impairment.

a Horowitz et al. (2005b, 2006) were derived from the same study cohort but were analyzed separately and examined different exposures with different study designs (cross-sectional vs longitudinal). To avoid double-counting, they were counted as one study with two reports.

b Statistical significance was defined as two-sided, *p*-value < .05 in a multivariable model. For continuous variables, results indicate the change per 1-unit increase in the respective score. Point estimates and their precision were reported as presented in the original studies. To facilitate evidence synthesis, factor directions were aligned across studies.

c Greater ADL limitations are categorized as a risk factor (originally reported as a higher ADL score being protective).

d Poor self-rated health is categorized as a risk factor (originally reported as better self-rated health being protective).

e Better acceptance of VI and positive attitude toward compensation are categorized as protective factors (originally reported as lower acceptance of VI and attitude toward compensation scores being risk).

f Greater self-reported visual difficulty is categorized as a risk factor (originally reported as a higher visual function score being protective).

g Living at home alone or living in a nursing home is categorized as a risk factor (originally reported as living at home with others being protective).

h A greater level of social support is categorized as a protective factor (originally reported as a lower level of social support being risk).

**Table 3:** Study characteristics and identified risk and protective factors associated with depressive symptoms in the included longitudinal reports (*n *= 4).

**Author(s) (publication year), country** ^a^	Sample characteristics	VI measures (VI definitions)	Depressive symptom measures and definitions (prevalence/incidence of depressive symptoms)	Identified risk factors, protective factors, and factors that did not reach statistical significance for depressive symptoms (*B*, *SE*)^b^	Type of multivariate analyses; covariates that were adjusted for in multivariable models
Horowitz et al. (2003), United States	*N *= 95, community-based vision rehabilitation service convenience sample; 65–89 years old, mean age 76.9 years, 56.8% female, causes of VI: NR; prospective cohort study, 2-year follow-up with two time points	Snellen chart (NR)	20-item CES-D ≥ 16 (33.7%/25.3% for the 2-year follow-up)^a^	*Risk factors:* Older age (years; *B* 0.29, *SE* 0.14), poor self-rated health^c^ (*B* −1.94, *SE* 0.97) *Protective factors:* Greater stability of friendship relations (*B* −6.60, *SE* 2.61), use of rehabilitation (yes vs no; *B* −7.82, *SE* 2.23) *Factors that did not reach statistical significance:* Marital status (married vs not married; *B* −2.77, *SE* 1.85), self-reported visual difficulty (*B* −1.11, *SE* 0.31), change in self-reported visual difficulty from baseline to the 2-year follow-up (*B* 1.85, *SE* 1.95), ADL/IADL limitations (*B* 1.52, *SE* 0.16), emotional boundedness (*B* −9.56, *SE* 0.19), family understanding condition (*B* −2.57, *SE* 1.99)	*Type of analyses:* linear regression analysis *Covariates:* age, marital status, self-rated health, self-reported visual difficulty, change in self-reported visual difficulty from baseline to the 2-year follow-up, baseline depression score, ADL/IADL limitations, emotional boundedness, family understanding condition, stability of friendship relations, use of rehabilitation
Horowitz et al. (2005a), United States	*N *= 95, community-based vision rehabilitation service convenience sample; 65–89 years old, mean age 76.9 years, 56.8% female, causes of VI: NR; prospective cohort study, 2-year follow-up with two time points	Snellen chart (NR)	20-item CES-D ≥ 16 (33.7%/ 25.3% for the 2-year follow-up)^a^	*Risk factors:* None *Protective factors:* Use of low vision service (yes vs no; *B* −50.12, *SE* 20.17), a greater number of optical aids (*B* −10.24, *SE* 0.58) *Factors that did not reach statistical significance:* Age (years; *B* 0.16, *SE* 0.14), gender (female vs male; *B* −0.84, *SE* 10.69), self-reported visual difficulty (*B* −0.14, *SE* 0.31), self-rated health (*B* −10.79, *SE* 0.97), ADL/IADL limitations (*B* −0.01, *SE* 0.17), use of skill training (yes vs no; *B* −10.98, *SE* 20.14), use of counseling (yes vs no; *B* −20.95, *SE* 20.47), number of adaptive aids (*B* 0.39, *SE* 0.52)	*Type of analyses:* linear regression analysis *Covariates:* age, gender, self-rated health, self-reported visual difficulty, baseline depression score, ADL/IADL limitations, use of low vision service, use of skill training, use of counseling, use of optical aids, use of adaptive aids
Horowitz et al. (2006), United States	*N *= 438, community-based vision rehabilitation service convenience sample; 65–99 years old, mean age 80.4 years (*SD *= 7.4), 53.4% female, causes of VI: AMD 69.7%, cataract 37.1%, glaucoma 26.8%, DR 13.7%; prospective cohort study, 6-month follow-up with two time points	Snellen chart (NR)	20-item CES-D, definition was NR (NR/NR)	*Risk factors:* Greater ADL/IADL limitations (*B* 0.10, *SE* 0.05) *Protective factors:* A greater number of optical aids at the 6-month follow-up (*B* −0.90, *SE* 0.43) *Factors that did not reach statistical significance:* Age (years; *B* −0.09, *SE* 0.05), gender (female vs male; *B* 0.09, *SE* 0.67), race (non-Hispanic White vs other races; *B* 1.57, *SE* 1.05), education (years; *B* 0.12, *SE* 0.21), total hours of rehabilitation services (hours; *B* 0.02, *SE* 0.04), number of adaptive aids at the 6-month follow-up (*B* −0.26, *SE* 0.24)	*Type of analyses:* linear regression analysis *Covariates:* age, gender, race, education, baseline depression score, ADL/IADL limitations, total hours of rehabilitation services, number of optical aids at the 6-month follow-up, number of adaptive aids at the 6-month follow-up
Schilling et al. (2016), United States	*N *= 364, community-based vision rehabilitation service convenience sample; 65–98 years old, mean age 83.0 years, 63.0% female, causes of VI: AMD 100.0%; prospective cohort study, 2-year follow-up with five time points	Snellen chart (BCVA 20/60 or worse)	20-item CES-D, definition was NR (NR/NR)	*Risk factors:* Older age (years; *B* 0.03, *SE* 0.01) *Protective factors:* Female sex (female vs male; *B* −0.22, *SE* 0.09), greater selective primary control strategies (*B* −0.40, *SE* 0.15), greater compensatory secondary control strategies (*B* −0.27, *SE* 0.08) *Factors that did not reach statistical significance:* Compensatory Primary Control strategies (*B* 0.10, *SE* 0.08), Selective Secondary Control strategies (*B* −0.11, *SE* 0.11)	*Type of analyses:* generalized linear mixed models *Covariates:* age, sex, selective primary control strategies, Compensatory Primary Control strategies, Selective Secondary Control strategies, Compensatory Secondary Control strategies

*Note*. ADL = activities of daily living; AMD = age-related macular degeneration; *B* = unstandardized coefficient; BCVA = best-corrected visual acuity; CES-D = Center for Epidemiologic Studies Depression Scale; IADL = instrumental activities of daily living; NR = not reported; *SD* = standard deviation; *SE* = standard error; VI = visual impairment.

a Horowitz et al. (2003, 2005a) were derived from the same study cohort but were analyzed separately and examined different exposures. Horowitz et al. (2005b, 2006) were derived from the same study cohort but were analyzed separately and examined different exposures with different study designs (cross-sectional vs longitudinal). To avoid double-counting, they were counted as one study with two reports.

b Statistical significance was defined as two-sided, *p*-value < .05 in a multivariable model. For continuous variables, results indicate the change per 1-unit increase in the respective score. Point estimates and their precision were reported as presented in the original studies. To facilitate evidence synthesis, factor directions were aligned across studies.

c Poor self-rated health is categorized as a risk factor (originally reported as a higher self-rated health score being protective).

In total, 3,013 participants were represented in 13 unique studies, with sample sizes ranging from 53 to 584 participants. The mean age of the samples ranged from 72.6 to 83.7 years (standard deviation [*SD*] range = 4.8–9.1). Three studies did not report the mean age for a total sample (Gouliopoulos et al., 2026; Ip et al., 2000; Zhou, 2025). Four studies targeted older adults aged 60 or older, whereas eight studies targeted aged 65 or older. The proportion of females/women ranged from 36.4% to 100.0%, with 11 studies reporting that ≥50% of participants were female. Among five studies that reported race/ethnicity, most were conducted in predominantly White populations, with three (Horowitz et al., 2005b, 2006; Kleinschmidt et al., 1995; Schilling et al., 2016) reporting that ≥80% of participants were White. Only one study (Horowitz et al., 2005b, 2006) included ≥10% Black or African American participants. Among nine studies that reported education, the most commonly reported level was at least high school completion, though the proportion varied widely across studies (6.6%–92.6%). Six of these studies reported that more than half of participants had completed at least a high school education. Supplementary Material Table 3 presents additional study characteristic variables reported in the included studies. Regarding the study settings, eight studies recruited from ophthalmologic clinics, four were from community-based vision rehabilitation services, and one from long-term care facilities. Overall, reporting of sociodemographic characteristics beyond age and sex/gender was limited and heterogeneous across all studies.

#### Physical function

Physical function was assessed in five studies, most commonly using activities of daily living (ADL) and instrumental activities of daily living (IADL) measures, such as the Older Americans Resources and Services (OARS) Multidimensional Functional Assessment Questionnaire and the Nottingham Extended ADL scale. Reported levels of functional limitation varied. Use of mobility aid (including white canes) was assessed in two studies (Horowitz et al., 2006; Palagyi et al., 2016).

#### Cognitive function

Seven studies excluded participants with cognitive impairment during screening. Cognitive impairment was assessed by the Mental Status Questionnaire, the Mini-Mental State Examination or the Six-Item Cognitive Impairment Test (Holloway et al., 2015; Horowitz et al., 2005b, 2006; Jivraj et al, 2013; Tolman et al., 2005). However, no included study reported cognitive function as an exposure measure or a covariate.

#### Comorbidity

The presence or/and number of comorbidities was reported in seven studies. The proportion of participants with at least one comorbidity ranged from 16.1% to 99.1%, and the mean number of comorbidities ranged from 2.2 to 4.0 (*SD* range = 1.4–2.0). The type of comorbidities was specified in three studies (Ip et al., 2000; Jivraj et al, 2013; Zhou, 2025), which reported conditions such as hypertension, dyslipidemia, dementia, and diabetes.

### Common theoretical frameworks

Only one study (Schilling et al., 2016) used at least one theoretical framework. The theory used in this study was the motivational theory of life-span development.

### Nomenclature for visual impairment

A total of 21 terms was identified to describe visual impairment in 15 included reports (Supplementary Material Table 4). Vision loss was the most common term used in 14 reports, followed by visual impairment in 12 reports and vision impairment in 11 reports. All included reports used at least two terms interchangeably to describe visual impairment. The median number of terms that were used in reports was 6 (range = 2–12).

**Table 4:** Recommendations for future research on depressive symptoms in older adults with visual impairment.

Topics	Recommendations
Definitions of visual impairment	Establish standardized definitions for visual impairment
Definitions of depressive symptoms	Determine clinically meaningful cutoff points for validated depressive symptoms measures
Measures of depressive symptoms	Compare self-reported and clinical evaluations (e.g., structured clinical interview for DSM disorders) Address potential measurement bias in interviewer-administered assessments Incorporate objective indicators (e.g., biomarkers, neuroimaging, genetic data)
Risk and protective factors associated with depressive symptoms	Examine associations with duration and change of visual impairment Examine associations with number and type of comorbidities Examine associations with social isolation/loneliness Examine associations with cognitive function/Alzheimer’s disease and related dementias Examine associations with stigma/discrimination related to visual impairment and disabilities Examine associations with access to mental health services Explore characteristics of type, severity, duration, history, and treatments of depressive symptoms Explore biological, environmental, and life-course determinants of depressive symptoms Examine psychosocial mechanisms linking visual impairment and depressive symptoms Examine longitudinal trajectories of depressive symptoms in relation to personal and environmental exposures
Generalizability	Include underrepresented populations (e.g., low- and middle-income countries, racial/ethnic minorities, low educational attainment) Include participants with chronic neurological conditions (e.g., dementia, Parkinson’s disease, stroke) Include participants in long-term and home care settings Use random sampling or nationally representative cohorts
Methodological rigor	Conduct a priori sample size calculations Report point estimates with 95% confidence intervals (not only *p*-values) Use longitudinal designs to establish temporality Ensure sufficient follow-up periods to assess effects of exposure and outcome Control for common confounders Apply theoretical frameworks to guide research and uncover underlying mechanisms

*Note*. DSM = Diagnostic and Statistical Manual of Mental Disorders.

Although multiple terms were used in the included reports, “visual impairment” was selected as the term for this review because it is widely used in gerontological and public health literature. Furthermore, while “vision loss” emphasizes the physiological loss of visual acuity, “visual impairment” encompasses a broader range of visual difficulties, including reductions in visual acuity, visual field, and related functional limitations, which is more consistent with the inclusive scope of this review.

### Common measures of visual impairment

The Snellen chart was the most common measure of visual impairment used to assess visual acuity in 10 of 13 studies, followed by the logMAR chart in four studies. One study used the Early Treatment Diabetic Retinopathy Study chart (Palagyi et al., 2016) and decimal visual acuity chart (Zhou, 2025).

### Common definitions of visual impairment

Of six studies that defined visual impairment, all studies used the cutoff score of visual acuity measures, using heterogeneous definitions of visual impairment. One study used visual acuity of 20/30 or worse measured by the Snellen chart (Yimer et al., 2021). Of the five studies assessed the best-corrected visual acuity (BCVA) measured by the Snellen chart, they defined visual impairment as BCVA 20/60 or worse (Schilling et al., 2016), BCVA 20/40 or worse in the better eye (Holloway et al., 2015), BCVA 20/80 or worse in the better eye (Hayman et al., 2007), BCVA 20/200 or worse in both eyes (Tolman et al., 2005), and BCVA worse than 20/400 in the better eye (Ip et al., 2000).

### Common causes of visual impairment

Causes of visual impairment were specified in 11 studies. The most common causes of visual impairment included age-related macular degeneration (51.2%–100.0%), cataract (2.4%–100%), glaucoma (17.9%–31.3%), and diabetic retinopathy (7.8%–14.7%). Seven studies recruited participants with a single cause of visual impairment, including older adults with age-related macular degeneration in five studies, and cataract in two studies.

### Common measures of depressive symptoms

The Geriatric Depression Scale (GDS) (*n *= 6) was the most common measure used to assess depressive symptoms, followed by the Center for Epidemiologic Studies Depression Scale (CES-D) (*n *= 4). In addition, two studies used the Zung Self-Rating Depression Scale (SDS), and one study used the Patient Health Questionnaire (PHQ). One study used the Structured Clinical Interview for DSM Disorders to determine a current diagnosis and past history of major depressive disorders (Horowitz et al., 2005b). All included studies used validated, self-reported measures to evaluate depressive symptoms.

### Common definitions of depressive symptoms

Of 11 studies that defined depressive symptoms, all studies used the cutoff score of depressive symptom measures as the definition of depressive symptoms. Three studies used the 20-item CES-D score ≥16 (Horowitz et al., 2003, 2005a, 2005b; Jivraj et al, 2013), and two used the 15-item GDS score ≥6 (Ip et al., 2000; Yimer et al., 2021) and the 20-item SDS score ≥50 (Gouliopoulos et al., 2026; Zhou, 2025). The remaining studies were defined depressive symptoms as the 5-item GDS score ≥2 (Palagyi et al., 2016), the 15-item GDS score >4 (Hayman et al., 2007), the 15-item GDS score ≥8 (Ip et al., 2000), the 30-item GDS score ≥11 (Kleinschmidt et al., 1995), and the 2-item PHQ score ≥3 (Holloway et al., 2015).

### Severity, duration, history, and treatment of depressive symptoms

Severity of depressive symptoms was reported in all 13 studies, although definitions and cutoffs varied widely, using the mean or categorical groups of depression measures. No study specified the duration of depressive symptoms. History of depressive symptoms was reported in four studies. Two studies excluded participants with prior history of depression diagnosis (Ip et al., 2000; Jivraj et al, 2013), while two studies included them (Holloway et al., 2015; Horowitz et al., 2005b). Treatments of depressive symptoms (e.g., antidepressant use) were specified in five studies.

### Prevalence and incidence of depressive symptoms

The 10 studies reported prevalence of depressive symptoms (Hayman et al., 2007; Holloway et al., 2015; Horowitz et al., 2003, 2005a, 2005b; Ip et al., 2000; Jivraj et al, 2013; Kleinschmidt et al., 1995; Palagyi et al., 2016; Yimer et al., 2021; Zhou, 2025). The mean prevalence of depressive symptoms was 31.4% (% range = 21.2%–44.7%). Only one study reported incidence of depressive symptoms (Horowitz et al., 2003, 2005a). The mean incidence of depressive symptoms was 25.3% at the 2-year follow-up, including 21.0% consistently depressed, 13.0% remitted depressive symptoms from baseline to follow-up, and 4.0% newly depressed at follow-up.

### Common covariates that were adjusted for in multivariable models

Age (*n *= 14) and sex/gender (*n *= 11) were most commonly adjusted in multivariable models, followed by severity of visual impairment (*n *= 6) and ADL/IADL status (*n *= 6) (Supplementary Material Table 5).

### Common risk and protective factors associated with depressive symptoms

**Figure 2:**
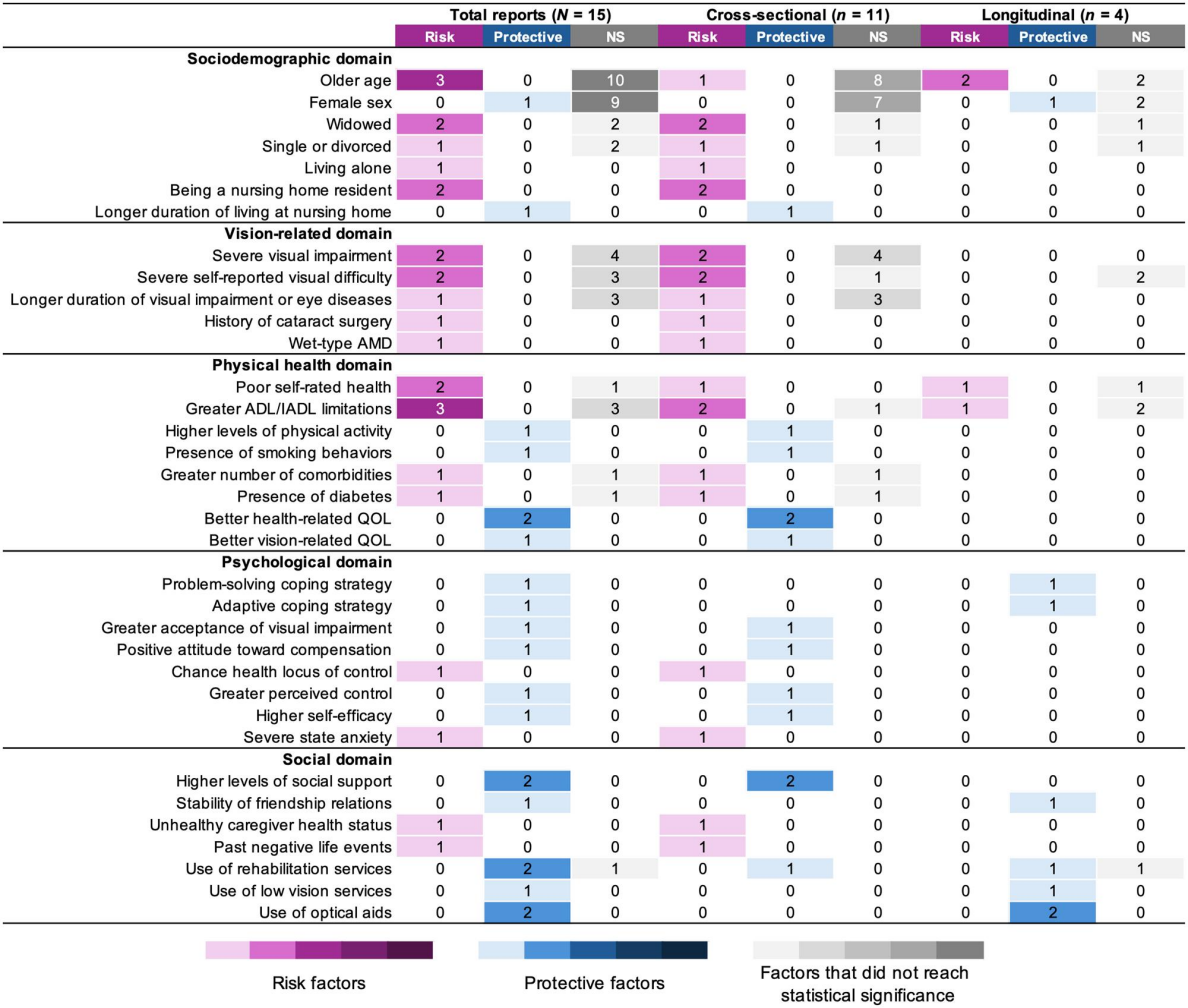
Heat map of risk and protective factors associated with depressive symptoms in older adults with visual impairment. *Note*. ADL = activities of daily living; AMD = age-related macular degeneration; IADL = instrumental activities of daily living; NS = factors that did not reach statistical significance; QOL = quality of life.

Tables 2 and 3 present statistically significant risk and protective factors associated with depressive symptoms among older adults with visual impairment using multivariable regression analyses. A heat map (Figure 2) visualized these factors clustered into sociodemographic, vision-related, physical health, psychological, and social domains. Across studies, depressive symptoms were modeled as continuous scores (i.e., unstandardized or standardized coefficients [*B* or beta] from linear or mixed-effects models) or binary outcomes (i.e., odds ratios or rate ratios from logistic or Poisson regression). For consistency, we describe factors as being associated with higher depressive symptoms (i.e., risk factors) or lower depressive symptoms (i.e., protective factors). Since Horowitz et al. (2003) and Horowitz et al. (2005a), as well as Horowitz et al. (2005b) and Horowitz et al. (2006), were derived from the same cohort but examined different exposures, each pair was counted as one study with two reports. Therefore, the numbers of identified risk factors, protective factors, and factors that did not reach statistical significance in the heat map were calculated based on 15 reports.

The identified factors associated with depressive symptoms were organized into five domains as follows. The sociodemographic domain included age, sex, marital status, and living arrangements. The vision-related domain included the severity of visual impairment, the severity of self-reported visual difficulty, and the duration of visual impairment or eye diseases. The physical health domain included self-rated health, ADL/IADL limitations, the number of comorbidities, physical activity, and health-related QOL. The psychological domain included coping strategies, acceptance of visual impairment, and self-efficacy. The social domain included social support and the use of rehabilitation services and optical aids.

#### Sociodemographic domain

##### Risk factors

Older age was significantly associated with higher depression scores or greater odds of depressive symptoms in three reports (Horowitz et al., 2003; Schilling et al., 2016; Zhou, 2025) but was not statistically significant in 10 reports (Gouliopoulos et al., 2026; Hayman et al., 2007; Holloway et al., 2015; Horowitz et al., 2005a, 2005b, 2006; Ip et al., 2000; Jivraj et al, 2013; Tolman et al., 2005; Yimer et al., 2021). Being widowed was also significantly associated with greater odds of depressive symptoms in two reports (Gouliopoulos et al., 2026; Yimer et al., 2021) but was not statistically significant in two reports (Horowitz et al., 2003, 2005b). Living alone (Jivraj et al, 2013) and being a nursing home resident (Hayman et al., 2007; Jivraj et al, 2013) were associated with higher depression scores or greater odds of depressive symptoms.

##### Protective factors

Female sex/gender was significantly associated with lower depressive symptom scores in one report (Schilling et al., 2016) but was not statistically significant in nine reports (Gouliopoulos et al., 2026; Hayman et al., 2007; Holloway et al., 2015; Horowitz et al., 2005a, 2005b, 2006; Jivraj et al, 2013; Tolman et al., 2005; Yimer et al., 2021). A longer duration of nursing home residence was also associated with lower odds of depressive symptoms (Ip et al., 2000).

##### Factors that did not reach statistical significance

Other sociodemographic factors, including race/ethnicity, education, financial status, and geographic areas, were not statistically significantly associated with depressive symptoms in multivariable analyses (Gouliopoulos et al., 2026; Hayman et al., 2007; Horowitz et al., 2005b, 2006; Tolman et al., 2005; Yimer et al., 2021).

#### Vision-related domain

##### Risk factors

Severe visual impairment was associated with greater odds of depressive symptoms in two reports (Holloway et al., 2015; Yimer et al., 2021) but was not statistically significant in four reports (Gouliopoulos et al., 2026; Hayman et al., 2007; Jivraj et al., 2013; Zhou, 2025). Severe self-reported visual difficulty was associated with higher depression scores or greater rates of depressive symptoms in two reports (Hayman et al., 2007; Palagyi et al., 2016) but was not statistically significant in three reports (Horowitz et al., 2003, 2005a, 2005b). A longer duration of visual impairment or eye diseases was associated with greater odds of depressive symptoms in one report (Yimer et al., 2021) but was not statistically significant in three reports (Gouliopoulos et al., 2026; Ip et al., 2000; Jivraj et al., 2013). A history of cataract surgery and wet-type age-related macular degeneration (Gouliopoulos et al., 2026) were also associated with greater odds of depressive symptoms.

##### Factors that did not reach statistical significance

Changes in self-reported visual difficulty were not statistically significantly associated with depressive symptoms in multivariable analyses in one study (Horowitz et al., 2003).

#### Physical health domain

##### Risk factors

Poor self-rated health was significantly associated with higher depression scores and greater odds of depressive symptoms in two reports (Horowitz et al., 2003, 2005b) but was not statistically significant in one report (Horowitz et al., 2005a). Greater ADL/IADL limitations were significantly associated with higher depression scores and greater odds of depressive symptoms in three reports (Horowitz et al., 2005b, 2006; Ip et al., 2000) but was not statistically significant in three reports (Hayman et al., 2007; Horowitz et al., 2003, 2005a). A greater number of comorbidities was significantly associated with greater rates of depressive symptoms in one report (Palagyi et al., 2016) but was not statistically significant in one report (Horowitz et al., 2005b). Presence of diabetes was significantly associated with greater rates of depressive symptoms in one report (Zhou, 2025) but was not statistically significant in one report (Gouliopoulos et al., 2026).

##### Protective factors

A greater level of physical activity (Hayman et al., 2007), presence of smoking behaviors (Gouliopoulos et al., 2026), better health-related QOL (Hayman et al., 2007; Palagyi et al., 2016), and better vision-related QOL (Jivraj et al., 2013) were associated with lower depression scores or odds/rates of depressive symptoms.

##### Factors that did not reach statistical significance

Other physical health factors, including falls efficacy, balance level, a history of hypertension, cancer, and depression treatment, substance use, a total number of medications, and the use of antidepressants and oral antioxidant supplements, were not statistically significantly associated with depressive symptoms in multivariable analyses (Gouliopoulos et al., 2026; Hayman et al., 2007; Holloway et al., 2015; Yimer et al., 2021).

#### Psychological domain

##### Risk factors

Chance health locus of control (measured by the 18-item Multidimensional Health Locus of Control-Form A; Kleinschmidt et al., 1995) and severe state anxiety (measured by the State-Trait Anxiety Inventory-6 Item [STAI-6]; Hayman et al., 2007) were associated with higher depression scores.

##### Protective factors

Problem-solving coping strategies (*operationalized as Selective Primary Control*, referring to investing one’s effort and time to achieve a goal, measured by the vision-specific version of the Optimization In Primary and Secondary Control Scale; Schilling et al., 2016), adaptive coping strategies (*operationalized as Compensatory Secondary Control*, referring to goal adjustments with self-protective thinking, measured by the vision-specific version of the Optimization In Primary and Secondary Control Scale; Schilling et al., 2016), greater acceptance of visual impairment (measured by the 24-item Adaptation to Vision Loss Scale; Tolman et al., 2005), positive attitude toward compensation (measured by the 24-item Adaptation to Vision Loss Scale; Tolman et al., 2005), greater perceived control (measured by the 16-item Desired Control Measure-Part II-Short Form; Kleinschmidt et al., 1995), and higher self-efficacy (measured by the 9-item Self-efficacy Scale; Horowitz et al., 2005b) were associated with lower depression scores or odds of depressive symptoms.

##### Factors that did not reach statistical significance

Other psychological factors, including alternative coping strategies (e.g., Compensatory Primary or Selective Secondary Control), other dimensions of health locus of control (e.g., powerful others, internal), negative impact on relationships, and perceived need for psychological help, were not statistically significantly associated with depressive symptoms in multivariable analyses (Holloway et al., 2015; Kleinschmidt et al., 1995; Schilling et al., 2016).

#### Social domain

##### Risk factors

Unhealthy caregiver health status (Zhou, 2025) and past negative life events (Horowitz et al., 2005b) were associated with greater odds of depressive symptoms.

##### Protective factors

The use of rehabilitation services was significantly associated with lower depression scores in two reports (Horowitz et al., 2003; Tolman et al., 2005), but total hours of rehabilitation services were not statistically significant in one report (Horowitz et al., 2006). A greater level of social supports (Horowitz et al., 2005b; Yimer et al., 2021), stability of friendship relationships (Horowitz et al., 2003), and the use of low vision services (Horowitz et al., 2005a) and optical aids (Horowitz et al., 2005a, 2006) were associated with lower depression scores or odds of depressive symptoms.

##### Factors that did not reach statistical significance

Other social factors, including family understanding of condition, emotional boundedness, the use of skill training, counseling, and adaptive aids, were not statistically significantly associated with depressive symptoms in multivariable analyses (Horowitz et al., 2003, 2005a, 2006).

## Discussion

To the best of our knowledge, this is the first systematic review to integrate the evidence on risk and protective factors associated with depressive symptoms among older adults with visual impairment. Overall, findings suggest that the identified risk factors include older age, being widowed, being a nursing home resident, severe visual impairment, severe self-reported visual difficulty, poor self-rated health, and greater ADL/IADL limitations. Protective factors included better health-related QOL, better adaptive coping strategies, a greater level of social support, and the use of rehabilitation services and optical aids. Although some identified factors, such as older age and ADL/IADL limitations, are commonly associated with the risk of depression in the general older population (Maier et al., 2021; Zenebe et al., 2021), our findings highlight factors that are uniquely critical in the context of visual impairment, specifically severe visual impairment and severe self-reported visual difficulty. Moreover, better adaptive coping strategies, a greater level of social support, and the use of rehabilitation services emerged as addressable protective factors, underscoring the importance of vision-specific interventions. These findings provide clinicians, researchers, and policymakers with data that enhance their understanding of modifiable risks and protective factors associated with depressive symptoms and can serve to underpin the development of targeted interventions designed to prevent depressive symptoms in older adults with visual impairment.

Across the 15 reports, no single factor demonstrated statistical significance in four or more reports. This lack of consistency highlights a need for study replication. Furthermore, only three of the 15 reports received a “good” quality rating, suggesting that the overall evidence is too limited to draw definitive conclusions. Therefore, the identified factors associated with depressive symptoms must be interpreted with caution, as the findings underscore the necessity for more rigorous future research.

Greater ADL/IADL limitations emerged as the most frequent risk factor associated with depressive symptoms in this review (three reports; Horowitz et al., 2005b, 2006; Ip et al., 2000). The association between functional limitations and depressive symptoms is aligned with prior quantitative studies in general older adults (Kiely et al., 2013; Weyerer et al., 2013) as well as older adults with visual impairment (Gong et al., 2020; Zhao et al., 2025). These findings are theoretically consistent with the DPM (Verbrugge & Jette, 1994), whereby visual impairment is associated with functional limitations in ADLs/IADLs, which may be associated with depressive symptoms. For example, visual impairment inhibits significant parts of IADLs, including driving, reading newspapers, and shopping at supermarkets. Prior qualitative studies (Bian et al., 2018; Dillon et al., 2021) found that older adults with visual impairment experience low self-esteem and dependence on others to assist with IADLs and ADLs, which may be associated with the development of depressive symptoms. Furthermore, after experiencing functional limitations, older adults with visual impairment tend to stay at home and restrict social interactions, which may contribute to social isolation (Bian et al., 2018; Dillon et al., 2021). Social isolation is known as a mediator between visual impairment and depressive symptoms among older adults (Gong et al., 2020; He et al., 2022). Future studies are needed to understand the relationship between visual impairment, functional limitations, social isolation, and depressive symptoms. Early detection of ADL/IADL decline and interventions to maintain ADL/IADL functions using low-vision rehabilitation and assistive aids could help prevent depressive symptoms.

In the sociodemographic domain, older age (Horowitz et al., 2003; Schilling et al., 2016) appeared as a potential risk factor. A prior systematic review in older adults also found consistent evidence that older age is associated with increased risk of depressive symptoms (Maier et al., 2021). However, in this review, most included reports did not reach statistical significance with depression scores, suggesting the evidence remains inconsistent. One included report (Horowitz et al., 2005b) showed that older eligible participants had a higher rate of refusal before enrollment. This indicates selective survival bias among study participants because those who are most vulnerable may not enroll or withdraw from the study. Therefore, the findings on age should be interpreted with caution. Age may act as a proxy for cumulative functional decline, as older age is associated with increased risk of poor mobility (Onyeso et al., 2026) and greater limitations in ADL and IADL (Yau et al., 2022), which are known risk factors for depressive symptoms among older adults. Future longitudinal studies are warranted to clarify whether age itself or functional disabilities explain the elevated risk of depressive symptoms in this population.

In the vision-related domain, severe visual impairment (Holloway et al., 2015; Yimer et al., 2021) and severe self-reported visual difficulty (Hayman et al., 2007; Palagyi et al., 2016) emerged as potential risk factors unique to older adults with visual impairment. Although evidence is limited, these findings theoretically align with the DPM (Verbrugge & Jette, 1994), where worsening functional impairments contribute to disabilities and subsequent depressive symptoms. There is mixed evidence regarding the association between the severity of visual impairment and depressive symptoms among older adults. One cohort study (Zhang et al., 2022) reported that poorer visual acuity was associated with higher odds of depressive symptoms. In contrast, one meta-analysis (Parravano et al., 2021) found that the prevalence of depressive symptoms did not vary by the severity of visual impairment. This inconsistency may reflect heterogeneity in study designs and measures of visual impairment across studies, which emphasizes the need for standardized approaches in future research. Our findings highlight the importance of focusing on screening depressive symptoms in high-risk groups, specifically older adults with severe visual impairment and self-reported visual difficulty, regardless of the causes of visual impairment. Co-locating interprofessional care that includes vision and mental health clinics in one setting may help with preventative screening of both vision and mental health status among older adults. Such integrated care models may be a promising approach to reduce barriers to mental healthcare access and facilitate early detection of depressive symptoms in ophthalmologic and low vision rehabilitation settings.

In the psychological domain, problem-solving coping strategies (i.e., investing one’s effort and time to achieve a goal; Schilling et al., 2016), adaptive coping strategies (i.e., goal adjustments with self-protective thinking; Schilling et al., 2016), greater acceptance of visual impairment (Tolman et al., 2005), and positive attitude toward compensation (Tolman et al., 2005) emerged as potential protective factors. Whereas the evidence for each factor is limited, the findings consistently suggest that greater problem-solving and adaptive coping strategies, greater acceptance of visual impairment, and positive attitude toward compensation serve as vital buffers against depressive symptoms. The finding regarding problem-solving coping strategies (Schilling et al., 2016) theoretically aligns with Lazarus and Folkman’s (1984) Stress and Coping Model, which involves cognitive appraisals and behavioral responses to manage a stressful situation, including visual impairment. Coping can be classified into problem-focused (i.e., directly managing a stressful situation) or emotion-focused coping (i.e., changing one’s emotional reactions from a stressful situation). Problem-focused coping encompasses brainstorming solutions, seeking information about the problem, and making plans to resolve a problem. Reinhardt et al. (2009) found that greater problem-solving coping strategies (i.e., take an action to improve the situation) were associated with reduced depression scores among older adults with visual impairment. This finding indicates that enhancing problem-solving coping strategies may help reduce the risk of depressive symptoms in this population. One clinical trial demonstrated that a program combining cognitive behavioral therapy and problem-solving treatment reduced the incidence of depressive symptoms among older adults with visual impairment (van der Aa et al., 2015). Further research is needed to identify optimal interventions to enhance problem-solving coping strategies and alleviate depressive symptoms in this population. Better adaptive coping strategies (Schilling et al., 2016), greater acceptance of visual impairment (Tolman et al., 2005), and positive attitude toward compensation (Tolman et al., 2005) theoretically align with Baltes and Baltes’ (1990) Selective Optimization with Compensation (SOC) Model, which involves prioritizing a specific goal (*Selection*), enhancing their remaining resources to achieve a goal (*Optimization*), and using substitutive resources to reach a goal (*Compensation*), to maximize gains and minimize losses in response to functional decline in later life. A prior empirical study also showed that better adaptation to age-related visual impairment buffers depressive symptoms among older adults (Heesterbeek et al., 2017). Similarly, other adaptive psychological resources, including optimism (Ji & Zhang, 2025) and psychological resilience (Górska et al., 2022), serve as buffers against depressive symptoms among older adults. The findings highlight the importance of focusing on an individual’s capability to psychologically adjust to late-life stressors, such as acquired visual impairment, and to recover from vision-related challenges, which can reduce the risk of depressive symptoms. Future research is needed to better understand the characteristics of adaptive psychological resources that help prevent and manage depressive symptoms in this population.

In the social domain, a greater level of social support (Horowitz et al., 2005b; Yimer et al., 2021), and the use of rehabilitation services (Horowitz et al., 2003; Tolman et al., 2005) and optical aids (Horowitz et al., 2005a, 2006) were identified as potential protective factors. The social domain encompasses external supports as well as access to and use of healthcare and rehabilitation services that may buffer vision-related distress. Specifically, optical aids were classified in this domain because they are typically provided through low vision rehabilitation services, reflecting healthcare service utilization rather than purely physical health or vision-related characteristics. These findings are theoretically consistent with the DPM (Verbrugge & Jette, 1994), which illustrates external supports, including personal assistants and assistive aids, as key factors that may buffer the impact of functional limitations on depressive symptoms. Prior studies have consistently shown that a greater level of social support is associated with lower depression scores among older adults with visual impairment (Gong et al., 2020; Reinhardt et al., 2009). Amilon and Siren (2022) further reported that emotional support mediated the association between visual impairment and lower depressive symptoms among adults. Therefore, approaches to enhancing access to social support are essential to mitigate the vision-related psychological distress. For example, Zhang et al. (2023) showed that frequent video calls with family or friends using digital apps (e.g., FaceTime, Zoom), which may facilitate emotional support to show empathy and acknowledge their feelings, reduced the likelihood of depressive symptoms in older adults with visual impairment during the COVID-19 pandemic. These findings suggest that enhancing access to and use of communication technologies (e.g., smartphone) represents a modifiable intervention to improve social support and reduce depressive symptoms. Evidence on rehabilitation services and optical aids is more mixed. A systematic review suggested that tailored interventions with group self-management techniques and behavioral activation with low-vision rehabilitation may be effective in preventing depressive symptoms among patients with age-related macular degeneration (Senra et al., 2019). However, other meta-analyses reported that there is no statistically significant effect of low-vision rehabilitation on depressive symptoms in this population (Hamade et al., 2016; Trott et al., 2025). Despite these inconsistencies, the potential of low-vision rehabilitation and optical aids cannot be overlooked. To maintain functional independence, low-vision rehabilitation services and optical aids are vital to address psychological distress in older adults with visual impairment. Expanding access to low-vision rehabilitation services and optical aids may help reduce the risk of depressive symptoms.

Better health-related QOL (Hayman et al., 2007; Palagyi et al., 2016) and vision-related QOL (Jivraj et al., 2013) were associated with a lower risk of depressive symptoms. However, these findings must be interpreted with caution because all included studies were cross-sectional, and causality cannot be determined. Consistent with our results, a prior systematic review also reported that worse health-related or vision-related QOL was associated with more severe depressive symptoms among older adults with visual impairment (Renaud & Bédard, 2013). Moreover, depressive symptoms and QOL conceptually overlap, as a prior study noted that both capture similar constructs of subjective well-being and functional health (Hays & Fayers, 2021). For instance, both the 25-item National Eye Institute Visual Functioning Questionnaire (NEI-VFQ) (i.e., a vision-related QOL scale) (National Eye Institute, 2000), which measures mental health symptoms due to change in vision functioning (e.g., feel frustrated, reduced sense of accomplishment), and the 20-item CES-D (i.e., a depression scale) (Radloff, 1977), which assesses mood disturbance (e.g., depressed moods, reduced pleasure), encompass features of affective and psychological status. Owsley and McGwin (2004) found that higher CES-D scores were significantly associated with lower NEI-VFQ scores among older adults in a cross-sectional design, further supporting the conceptual overlap between depressive symptoms and QOL measures. Future longitudinal research with distinct temporality is needed to clarify whether QOL should be considered an outcome, an exposure on depressive symptoms, or should be independently examined as outcomes, in this population.

Surprisingly, no included studies reported cognitive function as an exposure measure or a covariate, and most studies excluded individuals with cognitive impairment during screening. This is a critical knowledge gap, given that both visual impairment and depression have been identified as potentially modifiable risk factors for dementia, according to the 2024 Lancet Commission Report (Livingston et al., 2024). New-onset late-life depression may be a prodromal symptom of dementia, and the link between depression and dementia is considered bidirectional (Huang et al., 2024; Livingston et al., 2024). Furthermore, the co-occurrence of depression and dementia may be attributed to shared risk factors, such as hypertension and physical inactivity (Huang et al., 2024; Maier et al., 2021). Future longitudinal research should incorporate cognitive assessment for a better understanding of the relationship between visual impairment, depressive symptoms, and dementia risk among middle-aged and older adults.

Our review proposed recommendations for future research based on limitations and methodological quality assessment in the included studies (Table 4). These knowledge gaps represent critical directions for advancing the understanding of modifiable risk and protective factors associated with depressive symptoms in older adults with visual impairment. First, the included studies used heterogeneous measures and definitions for visual impairment and depressive symptoms. Establishing standardized definitions for visual impairment and determining clinically meaningful cutoff points for depressive symptoms measures are needed to provide comparable evidence across studies. Future studies should also explore broader biological, environmental, and life-course determinants of depressive symptoms, which remain largely unexplored in this population. To improve generalizability, future research should include underrepresented populations (e.g., individuals in low- and middle-income countries, racial and ethnic minorities) as well as those in long-term and home care settings to expand the applicability of the evidence. Using random sampling or nationally representative cohort data can also help reduce the risk of selection bias. In terms of methodological challenges, only one study (Schilling et al., 2016) employed a theoretical framework to explain the relationship with depressive symptoms in this review. The lack of theoretical foundations may limit a comprehensive understanding of underlying relationships with depressive symptoms among older adults with visual impairment. Schilling et al. (2016) used the motivational theory of life-span development (Heckhausen & Schulz, 1995) to examine how the developmental regulation processes in everyday goal achievement affect depressive symptoms in this population. The application of theoretical frameworks can support a more comprehensive exploration of potential risk and protective factors associated with depressive symptoms. Future studies are warranted to apply theoretical frameworks to help uncover underlying mechanisms of depressive symptoms.

The findings provide useful implications for clinical practice, highlighting the need to routinely assess depressive symptoms in older adults with visual impairment, specifically among those with severe visual impairment or functional limitations. Integrating mental health screening into vision care clinics and low vision rehabilitation services may help identify high-risk individuals earlier. Moreover, enhancing adaptive coping strategies, access to social support, rehabilitation services, and optical aids may reduce the risk of depressive symptoms in this population. In terms of public health policy, the results underscore the importance of developing integrated care approaches that address both sensory and mental health needs in late life. Public health initiatives should prioritize equitable access to vision and mental health care services, specifically for underserved populations. These findings align with global frameworks such as the WHO’s Integrated Care for Older People Approach (ICOPE) (WHO, 2025b), which emphasize the need to strengthen intrinsic capacity in older adults. Introducing policies that integrate vision and mental health care may help prevent depressive symptoms and reduce healthcare and social costs.

This systematic review has several strengths. From a theoretical standpoint, this review makes a novel contribution by synthesizing both risk and protective factors associated with depressive symptoms in older adults with visual impairment across sociodemographic, vision-related, physical health, psychological, and social domains. Unlike the only prior review in this population (Ribeiro et al., 2015), which focused solely on physical health factors and did not restrict inclusion to studies that adjusted for multiple confounders, this review provides a more rigorous and holistic evidence foundation. The findings provide implications for clinical practice, public health policy, and theoretical frameworks on late-life depression. Methodologically, this review also demonstrates several strengths. First, it follows the PRISMA 2020 statement, which enhances the rigor and transparency of the findings. Second, collaboration with three independent reviewers and two medical librarians helped reduce the risk of selection bias. Third, providing sample search strategies across five electronic databases and registering the protocol on PROSPERO improved reproducibility. Fourth, we included only quantitative studies that controlled for covariates of depressive symptoms using multivariable models, which helped enhance the rigor of synthesizing evidence. Overall, these strengths contribute to the methodological quality of this review.

Despite these strengths, several limitations should be acknowledged. First, the publication languages were limited to English, which may have introduced selection bias. Second, the findings are generalizable only to older adults aged 60 or older with visual impairment and may not extend to younger adults or those with dual sensory impairment or other types of visual difficulty (e.g., contrast sensitivity). Additionally, there were only 13 unique datasets used across the 15 reports which further limits generalizability. Third, while the reviewers developed a comprehensive search strategy across multiple databases with clear eligibility criteria, the exclusion of gray literature may have resulted in publication bias. Fourth, a meta-analysis could not be conducted due to substantial heterogeneity in definitions and measures of visual impairment and depressive symptoms. Since our qualitative evidence synthesis primarily relied on statistical significance (two-sided, *p*-value < .05), we may overlook clinically important associations that did not reach statistical significance. Fifth, residual confounding may have remained due to heterogeneous covariates controlled for in multivariable models. Finally, studies that assessed only self-reported visual difficulties other than objective visual acuity or that defined exposure as the presence of visual impairment were excluded. Therefore, potentially relevant risk and protective factors may have been omitted from the excluded studies.

In conclusion, this systematic review identified risk and protective factors associated with depressive symptoms among older adults with visual impairment. The identified risk factors were older age, being widowed, being a nursing home resident, severe visual impairment, severe self-reported visual difficulty, poor self-rated health, and greater ADL/IADL limitations. Protective factors included better health-related QOL, better adaptive coping strategies, a greater level of social support, and the use of rehabilitation services and optical aids. Our review underscores the importance of focusing on vision-specific risk factors as well as addressing the modifiable protective factors, such as adaptive coping strategies and social support. The findings imply the necessity of regular assessments to identify high-risk populations. Integrated care models that bridge aging, vision, and mental health services are essential to address the unique psychosocial needs of this population. Further studies are warranted to clarify the underlying mechanisms and to inform the design of personalized preventative approaches for late-life depression.

## Supplementary Material

gnag133_Supplementary_Data

## Data Availability

The protocol of this systematic review was registered with PROSPERO (ID: CRD42024602520).
