# Supplementary material for: Risk and protective factors associated with depressive symptoms in older adults with visual impairment: a systematic review
Source: Gerontologist. 2026 Jun 13;66(8):gnag133. doi: 10.1093/geront/gnag133 (PMC13372671; doi:10.1093/geront/gnag133)
Supplement: gnag133_Supplementary_Data [file gnag133_supplementary_data.zip › 01-Jul-2026_024650_H.Suzuki_et_al.Suppl.docx]

**Title Page**

**Title:** Risk and Protective Factors Associated With Depressive Symptoms in Older Adults With Visual Impairment: A Systematic Review

**Authors:** Haruno Suzuki, Heather Leutwyler, Jacqueline M. Torres, Kenneth Covinsky, Margaret Wallhagen

**Author name and affiliations:**

Haruno Suzuki, MHS, RN

[Haruno.Suzuki@ucsf.edu](mailto:Haruno.Suzuki@ucsf.edu)

PhD student

Department of Physiological Nursing

University of California, San Francisco, CA 94143, United States

ORCID: 0009-0000-7401-5721

Heather Leutwyler, RN, PhD, FNP-BC, CNS, FAAN, FGSA

[Heather.Leutwyler@ucsf.edu](mailto:Heather.Leutwyler@ucsf.edu)

Professor

Department of Physiological Nursing

University of California, San Francisco, CA 94158, United States

ORCID: 0000-0003-2823-1823

Jacqueline M. Torres, PhD, MPH

[Jacqueline.Torres@ucsf.edu](mailto:Jacqueline.Torres@ucsf.edu)

Associate Professor

Department of Epidemiology and Biostatistics

University of California, San Francisco, CA 94158, United States

ORCID: 0000-0002-1579-6107

Kenneth Covinsky, MD, MPH

[Ken.Covinsky@ucsf.edu](mailto:Ken.Covinsky@ucsf.edu)

Professor

Division of Geriatrics

University of California, San Francisco, CA 94158, United States

ORCID: 0009-0006-6119-6176

Margaret Wallhagen, PhD, GNP-BC, AGSF, FGSA, FAAN

[meg.wallhagen@ucsf.edu](mailto:meg.wallhagen@ucsf.edu)

Professor

Department of Physiological Nursing

University of California, San Francisco, CA 94158, United States

ORCID: 0000-0002-4988-2337

**Corresponding author:**

Haruno Suzuki, MHS, RN

Department of Physiological Nursing, School of Nursing, University of California, San Francisco

Address: 490 Illinois Street, San Francisco, CA 94143, United States

Email address: [Haruno.Suzuki@ucsf.edu](mailto:Haruno.Suzuki@ucsf.edu)

**Funding**

None reported.

**Conflict of Interest**

We have no conflict of interest to declare.

**Data Availability**

The protocol of this systematic review was registered with PROSPERO (ID: CRD42024602520).

**Acknowledgement**

An earlier version of this paper was presented at the Annual Scientific Meeting of the Gerontological Society of America, Boston, MA, November 2025. The authors acknowledge Dr. Christine Miaskowski, RN, PhD, FAAN, Dr. Yoshimi Fukuoka, RN, PhD, FAAN, Ms. Peggy Tahir, MLIS, and Ms. Eileen Chen, MLIS, for their contributions to the development of the review protocol and search strategies, abstract/title and full-text screening, evidence synthesis, and quality assessment.

**Author Contribution Statement**

Haruno Suzuki: Conceptualization, Methodology, Investigation, Data Curation, Formal Analysis, Visualization, Writing – Original Draft, Writing – Review & Editing.

Heather Leutwyler: Supervision, Writing – Review & Editing.

Jacqueline M. Torres: Writing – Review & Editing.

Kenneth Covinsky: Writing – Review & Editing.

Margaret Wallhagen: Supervision, Writing – Review & Editing.

**Supplementary Appendix**

**Supplementary Appendix Table 1. PRISMA 2020 checklist**

| **Section and Topic** | **Item #** | **Checklist item** | **Location where item is reported** |
| --- | --- | --- | --- |
| **TITLE** | | |  |
| Title | 1 | Identify the report as a systematic review. | Title |
| **ABSTRACT** | | |  |
| Abstract | 2 | See the PRISMA 2020 for Abstracts checklist. | Abstract |
| **INTRODUCTION** | | |  |
| Rationale | 3 | Describe the rationale for the review in the context of existing knowledge. | 4-5^th^ paragraphs in Introduction |
| Objectives | 4 | Provide an explicit statement of the objective(s) or question(s) the review addresses. | 6^th^ paragraph in Introduction |
| **METHODS** | | |  |
| Eligibility criteria | 5 | Specify the inclusion and exclusion criteria for the review and how studies were grouped for the syntheses. | “Eligibility criteria” section and Table 1 in Methods |
| Information sources | 6 | Specify all databases, registers, websites, organisations, reference lists and other sources searched or consulted to identify studies. Specify the date when each source was last searched or consulted. | “Information sources and search strategy” section and Supplementary Appendix Table 1 in Methods |
| Search strategy | 7 | Present the full search strategies for all databases, registers and websites, including any filters and limits used. | “Information sources and search strategy” section and Supplementary Appendix Table 1 in Methods |
| Selection process | 8 | Specify the methods used to decide whether a study met the inclusion criteria of the review, including how many reviewers screened each record and each report retrieved, whether they worked independently, and if applicable, details of automation tools used in the process. | “Selection process” section in Methods |
| Data collection process | 9 | Specify the methods used to collect data from reports, including how many reviewers collected data from each report, whether they worked independently, any processes for obtaining or confirming data from study investigators, and if applicable, details of automation tools used in the process. | “Data collection process and data items” section in Methods |
| Data items | 10a | List and define all outcomes for which data were sought. Specify whether all results that were compatible with each outcome domain in each study were sought (e.g. for all measures, time points, analyses), and if not, the methods used to decide which results to collect. | “Data collection process and data items” section and Table 1 in Methods |
|  | 10b | List and define all other variables for which data were sought (e.g. participant and intervention characteristics, funding sources). Describe any assumptions made about any missing or unclear information. | “Data collection process and data items” section and Table 1 in Methods |
| Study risk of bias assessment | 11 | Specify the methods used to assess risk of bias in the included studies, including details of the tool(s) used, how many reviewers assessed each study and whether they worked independently, and if applicable, details of automation tools used in the process. | “Risk of bias in individual studies” section in Methods |
| Effect measures | 12 | Specify for each outcome the effect measure(s) (e.g. risk ratio, mean difference) used in the synthesis or presentation of results. | “Data synthesis” section in Methods |
| Synthesis methods | 13a | Describe the processes used to decide which studies were eligible for each synthesis (e.g. tabulating the study intervention characteristics and comparing against the planned groups for each synthesis (item #5)). | “Data collection process and data items” and “Data synthesis” sections in Methods |
|  | 13b | Describe any methods required to prepare the data for presentation or synthesis, such as handling of missing summary statistics, or data conversions. | Not applicable |
|  | 13c | Describe any methods used to tabulate or visually display results of individual studies and syntheses. | “Data synthesis” section in Methods |
|  | 13d | Describe any methods used to synthesize results and provide a rationale for the choice(s). If meta-analysis was performed, describe the model(s), method(s) to identify the presence and extent of statistical heterogeneity, and software package(s) used. | Not applicable |
|  | 13e | Describe any methods used to explore possible causes of heterogeneity among study results (e.g. subgroup analysis, meta-regression). | Not applicable |
|  | 13f | Describe any sensitivity analyses conducted to assess robustness of the synthesized results. | Not applicable |
| Reporting bias assessment | 14 | Describe any methods used to assess risk of bias due to missing results in a synthesis (arising from reporting biases). | Not applicable |
| Certainty assessment | 15 | Describe any methods used to assess certainty (or confidence) in the body of evidence for an outcome. | Not applicable |
| **RESULTS** | | |  |
| Study selection | 16a | Describe the results of the search and selection process, from the number of records identified in the search to the number of studies included in the review, ideally using a flow diagram. | “Study selection” section in Results |
|  | 16b | Cite studies that might appear to meet the inclusion criteria, but which were excluded, and explain why they were excluded. | “Study selection” section in Results and Figure 1 |
| Study characteristics | 17 | Cite each included study and present its characteristics. | “Study characteristics” section in Results |
| Risk of bias in studies | 18 | Present assessments of risk of bias for each included study. | “Risk of bias in individual studies” section in Results |
| Results of individual studies | 19 | For all outcomes, present, for each study: (a) summary statistics for each group (where appropriate) and (b) an effect estimate and its precision (e.g. confidence/credible interval), ideally using structured tables or plots. | Table 2-3 |
| Results of syntheses | 20a | For each synthesis, briefly summarise the characteristics and risk of bias among contributing studies. | “Common risk and protective factors associated with depressive symptoms” and “risk of bias in individual studies” sections in Results |
|  | 20b | Present results of all statistical syntheses conducted. If meta-analysis was done, present for each the summary estimate and its precision (e.g. confidence/credible interval) and measures of statistical heterogeneity. If comparing groups, describe the direction of the effect. | Not applicable |
|  | 20c | Present results of all investigations of possible causes of heterogeneity among study results. | Not applicable |
|  | 20d | Present results of all sensitivity analyses conducted to assess the robustness of the synthesized results. | Not applicable |
| Reporting biases | 21 | Present assessments of risk of bias due to missing results (arising from reporting biases) for each synthesis assessed. | Not applicable |
| Certainty of evidence | 22 | Present assessments of certainty (or confidence) in the body of evidence for each outcome assessed. | Not applicable |
| **DISCUSSION** | | |  |
| Discussion | 23a | Provide a general interpretation of the results in the context of other evidence. | 1^st^-8^th^ paragraphs in Discussion |
|  | 23b | Discuss any limitations of the evidence included in the review. | 9^th^ paragraph in Discussion |
|  | 23c | Discuss any limitations of the review processes used. | 12^th^ paragraph in Discussion |
|  | 23d | Discuss implications of the results for practice, policy, and future research. | 10^th^ paragraph in Discussion |
| **OTHER INFORMATION** | | |  |
| Registration and protocol | 24a | Provide registration information for the review, including register name and registration number, or state that the review was not registered. | 1^st^ paragraph in Methods |
|  | 24b | Indicate where the review protocol can be accessed, or state that a protocol was not prepared. | 1^st^ paragraph in Methods |
|  | 24c | Describe and explain any amendments to information provided at registration or in the protocol. | 1^st^ paragraph in Methods |
| Support | 25 | Describe sources of financial or non-financial support for the review, and the role of the funders or sponsors in the review. | Title page |
| Competing interests | 26 | Declare any competing interests of review authors. | Title page |
| Availability of data, code and other materials | 27 | Report which of the following are publicly available and where they can be found: template data collection forms; data extracted from included studies; data used for all analyses; analytic code; any other materials used in the review. | Title page |

**Supplementary Appendix Table 2. Search strategies for PubMed, Embase, Web of Science, PsycINFO, and CINAHL Complete**

**PubMed**

The original search was conducted on November 17, 2024. The updated search using the same search strategy was conducted on November 20, 2025, to identify studies published between November 17, 2024, and November 20, 2025. The numbers of updated records included duplicates due to overlapping records on November 17, 2024. Duplicates were subsequently removed in Covidence during the screening process. No date limits were applied to the searches.

| **#** | **Search terms** | **Number of original records**  **(by Nov 17, 2024)** | **Number of updated records**  **(between Nov 17, 2024, and Nov 20, 2025)** |
| --- | --- | --- | --- |
| 1 | **Older adults**  old[tiab] OR older[tiab] OR aged[tiab] OR "Aged"[mh] OR aging[tiab] OR “senior citizens”[tiab] OR “senior adults”[tiab] OR elder[tiab] OR elders[tiab] OR elderly[tiab] OR geriatric[tiab] OR gerontology[tiab] OR gerontological[tiab] | 5,605,358 | 344,221 |
| 2 | **Visual impairment**  “visual impairment”[tiab] OR “vision impairment”[tiab] OR “visual disorder”[tiab] OR “visual disorders”[tiab] OR “vision disorder”[tiab] OR “vision disorders”[tiab] OR "Vision Disorders"[mh] OR “ocular impairment”[tiab] OR “ocular disorder”[tiab] OR “ocular disorders”[tiab] OR “vision loss”[tiab] OR “loss of vision”[tiab] OR “low vision”[tiab] OR "Vision, Low"[mh] OR “eye disease”[tiab] OR “eye diseases”[tiab] OR "Eye Diseases"[mh] OR "visually impaired"[tiab] OR "visually disabled"[tiab] OR "reduced vision"[tiab] OR "subnormal vision"[tiab] OR “sight loss”[tiab] OR “partially sighted”[tiab] OR "blindness"[tiab] OR "sensory loss"[tiab] OR glaucoma[tiab] OR "Glaucoma"[mh] OR "macular degeneration"[tiab] OR "Macular Degeneration"[mh] OR "diabetic retinopathy"[tiab] OR "Diabetic Retinopathy"[mh] | 722,730 | 26,281 |
| 3 | **Depression**  depression[tiab] OR “Depression”[mh] OR depressive[tiab] OR depressed[tiab] OR “Depressive Disorder"[mh] OR “treatment-resistant depressive disorder”[tiab] OR “treatment-resistant depressive disorders”[tiab] OR "Depressive Disorder, Treatment-Resistant"[mh] OR “bipolar disorder”[tiab] OR “bipolar disorders”[tiab] OR "Bipolar Disorder" [mh] OR “adjustment disorder”[tiab] OR “adjustment disorders”[tiab] OR "Adjustment Disorders"[mh] OR “affective disorder”[tiab] OR “affective disorders”[tiab] OR "Affective Disorders, Psychotic"[mh] | 670,250 | 46,173 |
| 4 | #1 AND #2 AND #3  Limited by English | 2,514 | 197 |
| **Total number of records on PubMed by November 20, 2025** | | **2,711** | |

**Embase**

The original search was conducted on November 17, 2024. The updated search using the same search strategy was conducted on November 20, 2025, to identify studies published between November 17, 2024, and November 20, 2025. The numbers of updated records included duplicates due to overlapping records on November 17, 2024. Duplicates were subsequently removed in Covidence during the screening process. No date limits were applied to the searches.

| **#** | **Search terms** | **Number of original records**  **(by Nov 17, 2024)** | **Number of updated records**  **(between Nov 17, 2024, and Nov 20, 2025)** |
| --- | --- | --- | --- |
| 1 | **Older adults**  'old':ti,ab OR 'older':ti,ab OR 'older adults’/exp OR 'aged':ti,ab OR 'aged'/exp OR 'aging':ti,ab OR 'aging'/exp OR 'senior citizens':ti,ab OR 'senior adults':ti,ab OR 'elder':ti,ab OR 'elders':ti,ab OR 'elderly':ti,ab OR 'geriatric':ti,ab OR 'geriatric'/exp OR 'gerontology':ti,ab OR 'gerontology’/exp OR 'gerontological':ti,ab | 6,992,409 | 745,354 |
| 2 | **Visual impairment**  'visual impairment':ti,ab OR 'vision impairment':ti,ab OR 'visual disorder':ti,ab OR 'visual disorders':ti,ab OR 'vision disorder':ti,ab OR 'vision disorders':ti,ab OR 'visual disorder'/exp OR 'ocular impairment':ti,ab OR 'ocular disorder':ti,ab OR 'ocular disorders':ti,ab OR 'vision loss':ti,ab OR 'loss of vision':ti,ab OR 'low vision':ti,ab OR 'low vision'/exp OR 'eye disease':ti,ab OR 'eye diseases':ti,ab OR 'eye disease'/exp OR 'visually impaired':ti,ab OR 'visually disabled':ti,ab OR 'reduced vision':ti,ab OR 'subnormal vision':ti,ab OR 'sight loss':ti,ab OR 'partially sighted':ti,ab OR 'blindness':ti,ab OR 'sensory loss':ti,ab OR 'glaucoma':ti,ab OR 'glaucoma'/exp OR 'macular degeneration':ti,ab OR 'macular degeneration'/exp OR 'diabetic retinopathy':ti,ab OR 'diabetic retinopathy'/exp | 1,295,121 | 102,840 |
| 3 | **Depression**  'depression':ti,ab OR 'depression'/exp OR 'depressive':ti,ab OR 'depressed':ti,ab OR 'treatment-resistant depressive disorder':ti,ab OR 'treatment-resistant depressive disorders':ti,ab OR 'treatment resistant depression'/exp OR 'bipolar disorder':ti,ab OR 'bipolar disorders':ti,ab OR 'bipolar disorder'/exp OR 'adjustment disorder':ti,ab OR 'adjustment disorders':ti,ab OR 'adjustment disorder'/exp OR 'affective disorder':ti,ab OR 'affective disorders':ti,ab OR 'affective psychosis'/exp | 1,034,990 | 110,600 |
| 4 | **Journal article or journal article in press**  'article'/it OR 'article in press'/it | 30,978,493 | 1,519,163 |
| 5 | #1 AND #2 AND #3 AND #4  Limited by English | 7,418 | 774 |
| **Total number of records on Embase by November 20, 2025** | | **8,192** | |

**Web of Science**

The original search was conducted on November 17, 2024. The updated search using the same search strategy was conducted on November 20, 2025, to identify studies published between November 17, 2024, and November 20, 2025. The numbers of updated records included duplicates due to overlapping records on November 17, 2024. Duplicates were subsequently removed in Covidence during the screening process. No date limits were applied to the searches.

| **#** | **Search terms** | **Number of original records**  **(by Nov 17, 2024)** | **Number of updated records**  **(between Nov 17, 2024, and Nov 20, 2025)** |
| --- | --- | --- | --- |
| 1 | **Older adults**  old OR older OR aged OR aging OR “senior citizens” OR “senior adults” OR elder OR elders OR elderly OR geriatric OR gerontology OR gerontological | 6,787,422 | 396,591 |
| 2 | **Visual impairment**  “visual impairment” OR “vision impairment” OR “visual disorder” OR “visual disorders” OR “vision disorder” OR “vision disorders” OR “ocular impairment” OR “ocular disorder” OR “ocular disorders” OR “vision loss” OR “loss of vision” OR “low vision” OR “eye disease” OR “eye diseases” OR "visually impaired" OR "visually disabled" OR "reduced vision" OR "subnormal vision" OR “sight loss” OR “partially sighted” OR "blindness" OR "sensory loss" OR glaucoma OR "macular degeneration" OR "diabetic retinopathy" | 289,628 | 18,020 |
| 3 | **Depression**  depression OR depressive OR depressed OR “bipolar disorder” OR “bipolar disorders” OR “adjustment disorder” OR “adjustment disorders” OR “affective disorder” OR “affective disorders” | 973,747 | 58,100 |
| 4 | #1 AND #2 AND #3  Limited by English | 2,324 | 173 |
| **Total number of records on Web of Science by November 20, 2025** | | **2,497** | |

**PsycINFO**

The original search was conducted on November 17, 2024. The updated search using the same search strategy was conducted on November 20, 2025, to identify studies published between November 17, 2024, and November 20, 2025. The numbers of updated records included duplicates due to overlapping records on November 17, 2024. Duplicates were subsequently removed in Covidence during the screening process. No date limits were applied to the searches.

| **#** | **Search terms** | **Number of original records**  **(by Nov 17, 2024)** | **Number of updated records**  **(between Nov 17, 2024, and Nov 20, 2025)** |
| --- | --- | --- | --- |
| 1 | **Older adults**  old OR older OR aged OR aging OR “senior citizens” OR “senior adults” OR elder OR elders OR elderly OR geriatric OR gerontology OR gerontological | 1,138,364 | 22,893 |
| 2 | **Visual impairment**  “visual impairment” OR “vision impairment” OR “visual disorder” OR “visual disorders” OR “vision disorder” OR “vision disorders” OR “ocular impairment” OR “ocular disorder” OR “ocular disorders” OR “vision loss” OR “loss of vision” OR “low vision” OR “eye disease” OR “eye diseases” OR "visually impaired" OR "visually disabled" OR "reduced vision" OR "subnormal vision" OR “sight loss” OR “partially sighted” OR "blindness" OR "sensory loss" OR glaucoma OR "macular degeneration" OR "diabetic retinopathy" | 26,515 | 494 |
| 3 | **Depression**  depression OR depressive OR depressed OR “bipolar disorder” OR “bipolar disorders” OR “adjustment disorder” OR “adjustment disorders” OR “affective disorder” OR “affective disorders” | 496,475 | 15,732 |
| 4 | #1 AND #2 AND #3  Limited by English | 799 | 20 |
| **Total number of records on PsycINFO by November 20, 2025** | | **819** | |

**CINAHL Complete**

The original search was conducted on November 17, 2024. The updated search using the same search strategy was conducted on November 20, 2025, to identify studies published between November 17, 2024, and November 20, 2025. The numbers of updated records included duplicates due to overlapping records on November 17, 2024. Duplicates were subsequently removed in Covidence during the screening process. No date limits were applied to the searches.

| **#** | **Search terms** | **Number of original records**  **(by Nov 17, 2024)** | **Number of updated records**  **(between Nov 17, 2024, and Nov 20, 2025)** |
| --- | --- | --- | --- |
| 1 | **Older adults**  old OR older OR aged OR aging OR “senior citizens” OR “senior adults” OR elder OR elders OR elderly OR geriatric OR gerontology OR gerontological | 1,366,047 | 59,192 |
| 2 | **Visual impairment**  “visual impairment” OR “vision impairment” OR “visual disorder” OR “visual disorders” OR “vision disorder” OR “vision disorders” OR “ocular impairment” OR “ocular disorder” OR “ocular disorders” OR “vision loss” OR “loss of vision” OR “low vision” OR “eye disease” OR “eye diseases” OR "visually impaired" OR "visually disabled" OR "reduced vision" OR "subnormal vision" OR “sight loss” OR “partially sighted” OR "blindness" OR "sensory loss" OR glaucoma OR "macular degeneration" OR "diabetic retinopathy" | 59,896 | 2,322 |
| 3 | **Depression**  depression OR depressive OR depressed OR “bipolar disorder” OR “bipolar disorders” OR “adjustment disorder” OR “adjustment disorders” OR “affective disorder” OR “affective disorders” | 249,199 | 14,424 |
| 4 | #1 AND #2 AND #3  Limited by English | 706 | 38 |
| **Total number of records on CINAHL Complete by November 20, 2025** | | **744** | |

**Supplementary Appendix Figure 1. Traffic light plot for the included cross-sectional reports (*n* = 11) to visualize risk of bias assessment by the National Heart, Lung, and Blood Institute’s (NHLBI) Quality Assessment Tool for Observational and Cross-sectional studies**


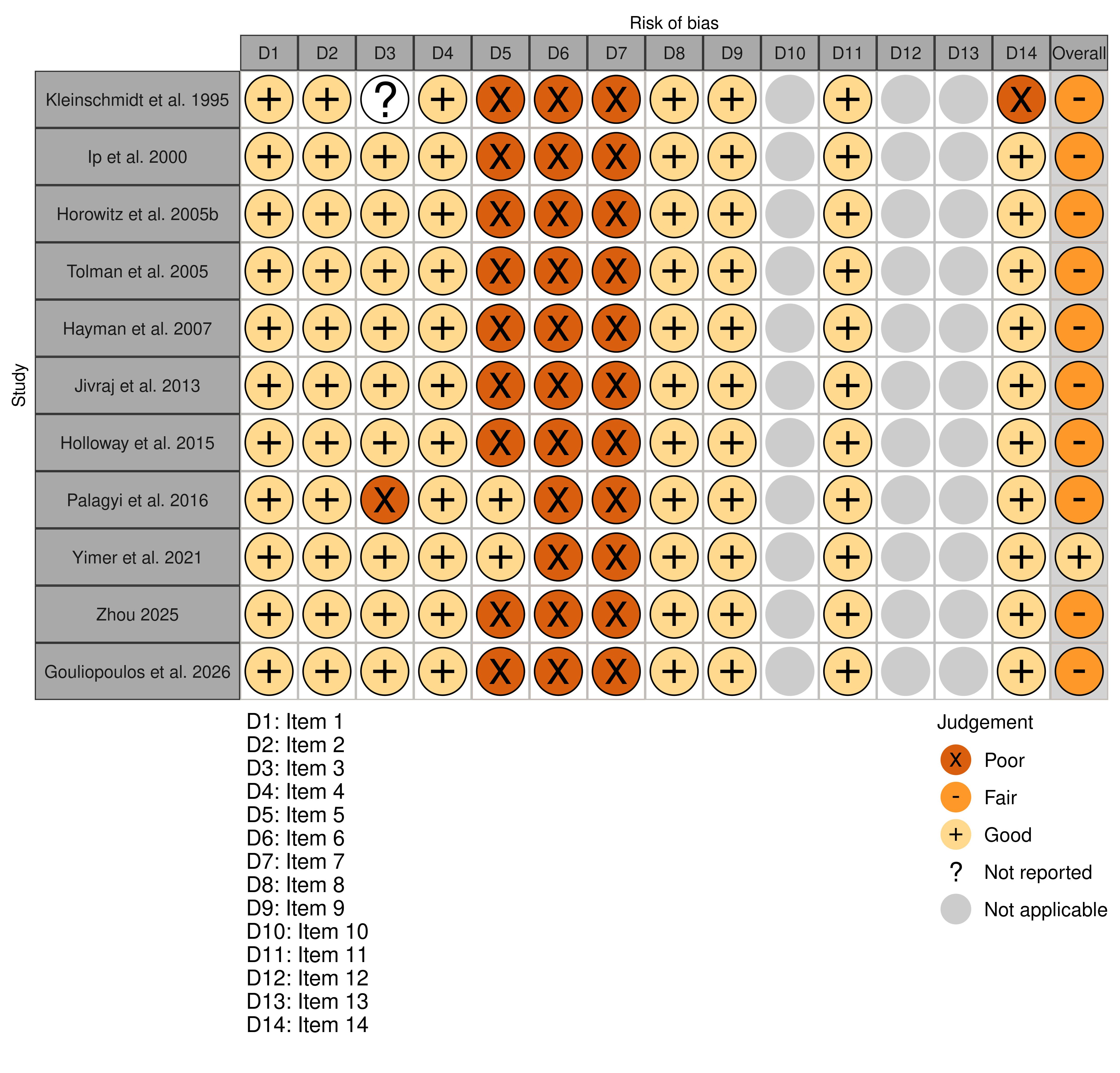

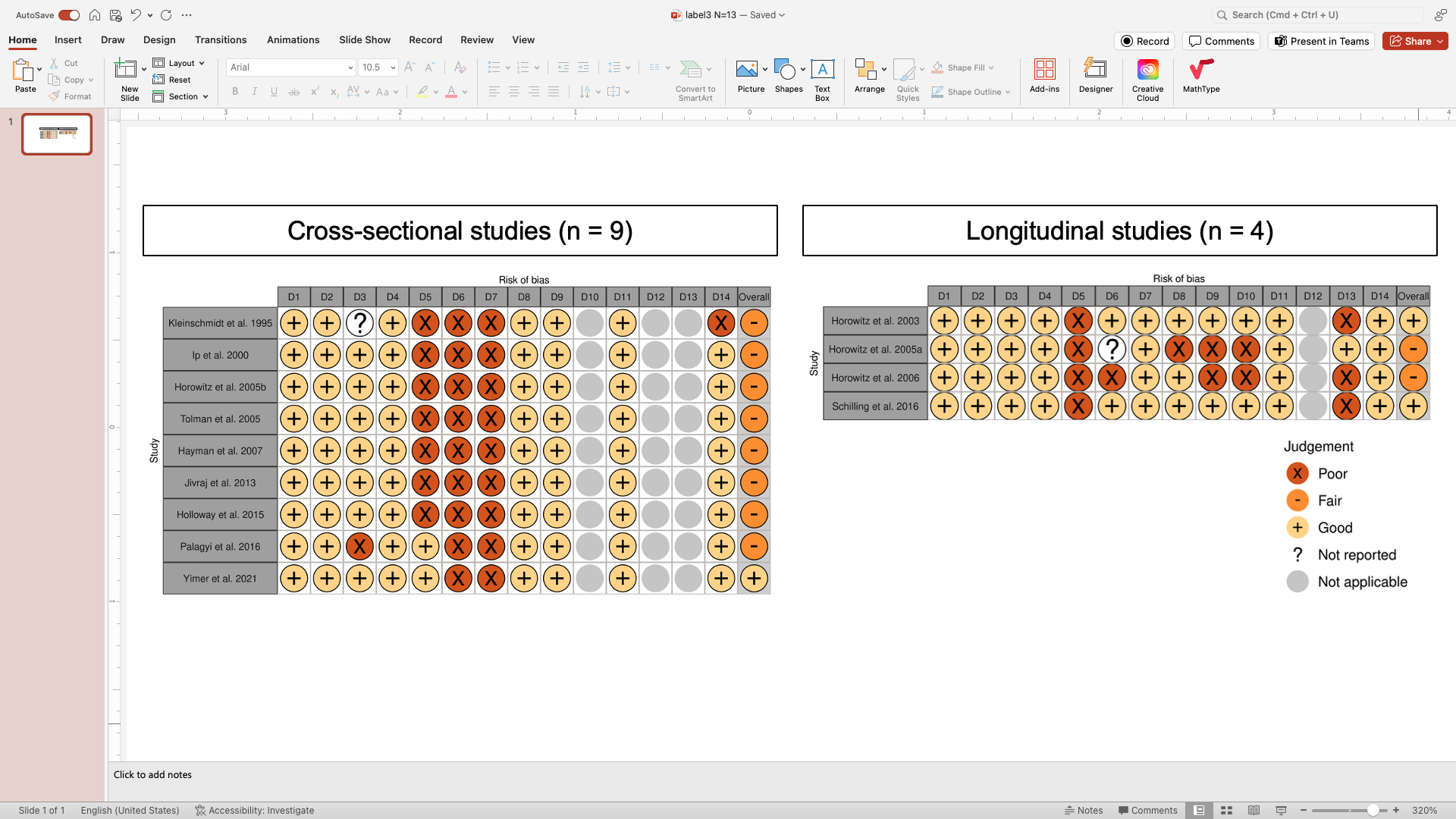


**Footnote.** NHLBI’s Quality Assessment Tool for Observational and Cross-sectional Studies Criteria: D1 (clear research question); D2 (define study population); D3 (participation rate at least 50%); D4 (uniform eligibility criteria); D5 (sample size justification); D6 (exposure assessed prior to outcome measurement); D7 (sufficient timeframe to see an effect); D8 (examine different levels of exposure); D9 (clearly defined and validated exposure measures); D10 (exposure assessed more than once over time); D11 (clearly defined outcome measures); D12 (outcome assessors were blinded to exposure status of participants); D13 (loss to follow-up less than 20%); D14 (key confounding variables measured and adjusted statistically). This traffic-light plot was created by robvis software using a colorblind-friendly palette.

**Supplementary Appendix Figure 2. Traffic light plot for the included longitudinal reports (*n* = 4) to visualize risk of bias assessment by the National Heart, Lung, and Blood Institute’s (NHLBI) Quality Assessment Tool for Observational and Cross-sectional studies**


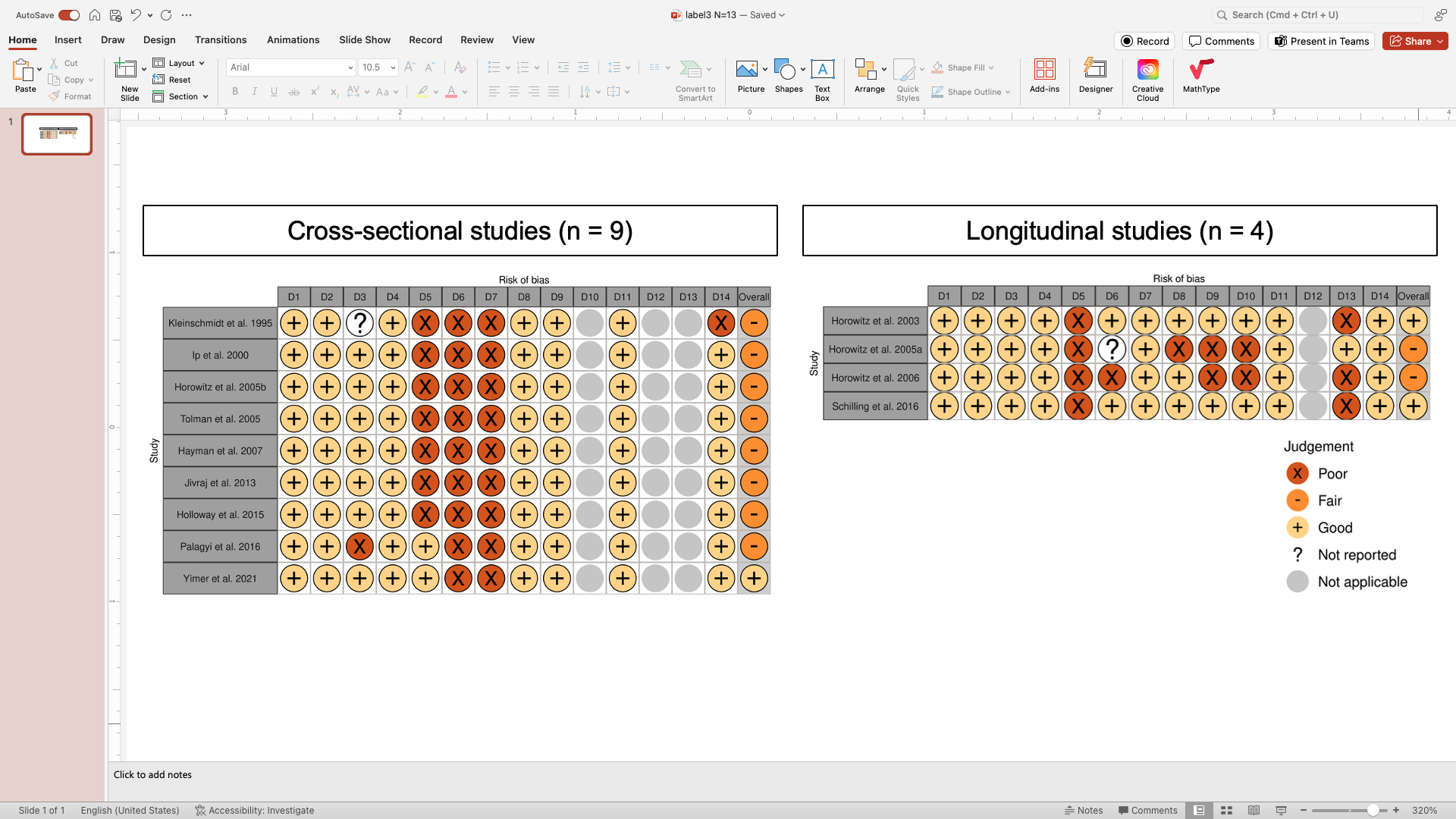


**Footnote.** NHLBI’s Quality Assessment Tool for Observational and Cross-sectional Studies Criteria: D1 (clear research question); D2 (define study population); D3 (participation rate at least 50%); D4 (uniform eligibility criteria); D5 (sample size justification); D6 (exposure assessed prior to outcome measurement); D7 (sufficient timeframe to see an effect); D8 (examine different levels of exposure); D9 (clearly defined and validated exposure measures); D10 (exposure assessed more than once over time); D11 (clearly defined outcome measures); D12 (outcome assessors were blinded to exposure status of participants); D13 (loss to follow-up less than 20%); D14 (key confounding variables measured and adjusted statistically). This traffic-light plot was created by robvis software using a colorblind-friendly palette.

**Supplementary Appendix Table 3. Study characteristic variables that were reported in 13 included studies**

| **Author(s) (publication year)** | **Age** | **Sex/**  **gender** | **Race/**  **ethnicity** | **Education** | **Income** | **Marital status** | **Living arrangement** | **Physical function^a^** | **Cognitive function^b^** | **Comorbidity^c^** |
| --- | --- | --- | --- | --- | --- | --- | --- | --- | --- | --- |
| Kleinschmidt et al. (1995) | Yes^d^ | Yes | Yes | Yes | Yes | Yes | No | No | No | No |
| Ip et al. (2000) | Yes | Yes | No | No | No | No | Yes | Yes | No | Yes |
| Horowitz et al. (2003, 2005a)^d^ | Yes | Yes | Yes | Yes | No | Yes | Yes | Yes | No | Yes |
| Horowitz et al. (2005b, 2006)^d^ | Yes | Yes | Yes | Yes | No | Yes | Yes | Yes | No | Yes |
| Tolman et al. (2005) | Yes | Yes | No | Yes | Yes | No | No | No | No | No |
| Hayman et al. (2007) | Yes | Yes | Yes | No | No | No | Yes | Yes | No | No |
| Jivraj et al. (2013) | Yes | Yes | No | Yes | No | No | Yes | No | No | Yes |
| Holloway et al. (2015) | Yes | Yes | No | Yes | No | Yes | No | No | No | Yes |
| Palagyi et al. (2016) | Yes | Yes | No | No | No | No | Yes | Yes | No | Yes |
| Schilling et al. (2016) | Yes | Yes | Yes | Yes | Yes | Yes | No | Yes | No | Yes |
| Yimer et al. (2021) | Yes | Yes | No | Yes | No | Yes | Yes | No | No | Yes |
| Zhou (2025) | Yes | Yes | No | Yes | Yes | Yes | No | No | No | Yes |
| Gouliopoulos et al. (2026) | Yes | Yes | No | Yes | No | Yes | No | No | No | Yes |
| **Total number of Yes^e^** | 13 | 13 | 5 | 10 | 4 | 8 | 7 | 6 | 0 | 10 |
| **Total number of No^e^** | 0 | 0 | 8 | 3 | 9 | 5 | 6 | 7 | 13 | 3 |

**Footnotes.**

^a^ Physical function includes reports of activities of daily living, instrumental activities of daily living, and/or use of mobility aids.

^b^ No included study reported cognitive function as an outcome measure or a covariate.

^c^ Comorbidity includes reports of presence, number, and/or type of comorbidity.

^d^ Horowitz et al. (2003, 2005a) were derived from the same study cohort but were analyzed separately and examined different exposures. Horowitz et al. (2005b, 2006) were derived from the same study cohort but were analyzed separately and examined different exposures with different study designs (cross-sectional vs. longitudinal). To avoid double-counting, they were counted as one study with two reports.

^e^ “Yes” means a variable that was reported in the included study. “No” means a variable that was not reported in the included study.

**Supplementary Appendix Table 4. Nomenclature to describe visual impairment in 15 included reports**

| **Common terms to describe visual impairment** | **Frequency (*n*)** |
| --- | --- |
| Vision loss | 14 |
| Visual impairment | 12 |
| Vision impairment | 11 |
| Blind | 9 |
| Visually impaired | 9 |
| Blindness | 8 |
| Vision problem | 7 |
| Legal blindness | 6 |
| Low vision | 6 |
| Loss of vision | 4 |
| Visual disability | 4 |
| Visual loss | 4 |
| Visually disabled | 3 |
| Impaired vision | 2 |
| Vision impaired | 2 |
| Vision-impaired | 2 |
| Visual limitation | 2 |
| Legally blind | 1 |
| Vision disability | 1 |
| Visual decline | 1 |
| Visual dysfunction | 1 |

**Supplementary Appendix Table 5. Common covariates that were adjusted for in multivariable regression models in 15 included reports**

| **Common covariates** | **Frequency (*n*)** |
| --- | --- |
| Age | 14 |
| Sex/gender | 11 |
| Severity of visual impairment | 6 |
| ADL/IADL status | 6 |
| Education | 5 |
| Self-report visual difficulty | 5 |
| Marital status | 4 |
| Duration of visual impairment | 4 |
| Race/ethnicity | 3 |
| Self-rated health | 3 |
| Baseline depression score | 3 |
| Use of rehabilitation services | 3 |
| Living arrangement | 2 |
| Geographic location | 2 |
| A history of diagnosed depression | 2 |
| A total number of chronic diseases | 2 |
| Presence of diabetes | 2 |
| Health-related QOL | 2 |
| Levels of social support | 2 |
| Use of optical aids | 2 |
| Use of adaptive aids | 2 |

**Abbreviations.** ADL – activities of daily living; IADL – instrumental activities of daily living; QOL – quality of life.
